# “All Polyimide” Mixed Matrix Membranes for High Performance Gas Separation

**DOI:** 10.3390/polym13081329

**Published:** 2021-04-19

**Authors:** Maijun Li, Zhibo Zheng, Zhiguang Zhang, Nanwen Li, Siwei Liu, Zhenguo Chi, Jiarui Xu, Yi Zhang

**Affiliations:** 1 PCFM Lab, GD HPPC Lab, Guangdong Engineering Technology Research Centre for High-performance Organic and Polymer Photoelectric Functional Films, State Key Laboratory of Optoelectronic Materials and Technologies, School of Chemistry, Sun Yat-sen University, Guangzhou 510275, China; limj7@mail2.sysu.edu.cn (M.L.); liusiw@mail.sysu.edu.cn (S.L.); chizhg@mail.sysu.edu.cn (Z.C.); xjr@mail.sysu.edu.cn (J.X.); 2 State Key Laboratory of Coal Conversion, Institute of Coal Chemistry, Chinese Academy of Sciences, Taiyuan 030001, China; zhangzhiguang@sxicc.ac.cn

**Keywords:** mixed-matrix membranes, compatibility, microporous polyimide particle, polyimide matrix, permeability

## Abstract

To improve the interfacial compatibility of mixed matrix membranes (MMMs) for gas separation, microporous polyimide particle (AP) was designed, synthesized, and introduced into intrinsic microporous polyimide matrix (6FDA-Durene) to form “all polyimide” MMMs. The AP fillers showed the feature of thermal stability, similar density with polyimide matrix, high porosity, high fractional free volume, large microporous dimension, and interpenetrating network architecture. As expected, the excellent interfacial compatibility between 6FDA-Durene and AP without obvious agglomeration even at a high AP loading of 10 wt.% was observed. As a result, the CO_2_ permeability coefficient of MMM with AP loading as low as 5 wt.% reaches up to 1291.13 Barrer, which is 2.58 times that of the pristine 6FDA-Durene membrane without the significant sacrificing of ideal selectivity of CO_2_/CH_4_. The improvement of permeability properties is much better than that of the previously reported MMMs, where high filler content is required to achieve a high permeability increase but usually leads to significant agglomeration or phase separation of fillers. It is believed that the excellent interfacial compatibility between the PI fillers and the PI matrix induce the effective utilization of porosity and free volume of AP fillers during gas transport. Thus, a higher diffusion coefficient of MMMs has been observed than that of the pristine PI membrane. Furthermore, the rigid polyimide fillers also result in the excellent anti-plasticization ability for CO_2_. The MMMs with a 10 wt.% AP loading shows a CO_2_ plasticization pressure of 300 psi.

## 1. Introduction

Membrane separation is the most promising gas separation technology with the advantages of low energy consumption, simple equipment, continuous operation at room temperature, and easy amplification [1,2]. Several classes of polymers have been used for gas separation. Among them, polyimides (PIs) based on 4,4′-(hexafluoroisopropylidene) diphthalic anhydride (6FDA) are emerging for various applications and are the most common used polymeric membranes due to their thermal stability, self-standing, acid resistance, and CO_2_ affinity [3,4,5,6]. However, PIs with higher selectivity generally have lower permeability and vice versa [7], which was first proposed by Robeson [8] and named as “trade-off” relationship [9].

To improve the gas separation performance of polymeric membrane, many efforts have been made to develop new membranes technologies, including the synthesis of polymers of intrinsic microporosity (PIM) [10], the post-treatment of polymeric membranes [11], the preparation of mixed-matrix membranes (MMMs) [12], etc. Among them, MMMs have attracted significant attention due to their versatility and effectivity. In general, MMMs are prepared by dispersing porous fillers such as activated carbon [13,14], kaolin [15], and metal–organic frameworks (MOFs) [16] into polymer matrix. Although the permeability and selectivity of MMMs can be potentially improved to some extent compared with those of the pure polymer membranes, the agglomeration of fillers due to the poor interfacial compatibility between the polymer and fillers limit their practical applications in gas separation [17,18]. 

Several approaches have been developed to improve the interfacial compatibility between polymer and fillers, including optimizing the mixing sequence of inorganic filler and polymer matrix, modification the fillers with functional groups, coating a thin layer onto inorganic fillers, using the microporous organic fillers, etc. For example, Zhu et al. [19] synthesized a MOF of [Cd_2_L(H_2_O)]_2_·5H_2_O (Cd-6F) using 4,4′-(hexafluoroisopropylidene)diphthalic anhydride (6FDA) as an organic ligand and introduced it into the 6FDA-ODA polyimide matrix to achieve novel MOF MMMs. They found that the MMM derived from in-situ polymerization presents excellent interfaces between micrometer-sized MOF crystals and the polymer matrix, resulting in increased permeability and selectivity. Zhu et al. [20] also coated a thin ionic liquid layer, which act as filler/matrix interfacial binder, onto MOF to prepare high compatible MMMs. Although improving the compatibility is helpful to improve the selectivity, the CO_2_ permeability ($P_{CO_{2}}$) decreased as the pores of MOF were blocked by ionic liquid. Rosi et al. [21] introduced UiO-66-NH_2_, a MOF with primary amine moiety which can interact with polymer matrix, into Matrimid to prepare MMM. They found that as high as a 23 wt.% of UiO-66-NH_2_ filling content was needed to achieve 2.5 times $P_{CO_{2}}\;$ increase. It is more difficult to modify when PI matrix has a high gas permeation performance. Smith et al. [21] dropped UiO-66-NH_2_ into polyimide of intrinsic microporosity matrix to prepare MMM. They found that 30 wt.% of UiO-66-NH_2_ filling content was needed to achieve the same $P_{CO_{2}}$ increment above. The above literature illustrates that modifying the inorganic filler will affect the gas permeation rate or high permeability can be obtained only under high inorganic filler content.

Microporous organic polymers (MOPs) with distorted molecular structure, which have the features of acid resistance, large microporous dimension, and interpenetrating network architecture, can be prepared by crosslink between triamine monomer and dianhydride monomer [22]. Because MOP and PI matrix are both amorphous, the dispersed phase and the continuous phase can bind to each other to make a defect-free interface. However, traditional MOPs can only modify low fractional free volume (FFV) matrix such as Matrimid because the porosity and FFV of traditional MOPs are not large enough [23]. 

In this work, three microporous PI particles, named AP, BP, and TP, were prepared by the reaction of tris(4-aminophenyl)amine (TAPA), 1,3,5-tris(4-nitrophenyl)benzene (TNPB), and 1,3,5-tris(4-aminophenyl)benzene (TAPB) with pyromellitic dianhydride (PMDA), respectively. The particles had a large amount of ultrafine micropores, high CO_2_ uptake, and high porosity and were used as fillers. The intrinsic microporous polyimide (6FDA-Durene), which is formed from 2,3,5,6-tetramethyl-1,4-phenylenediamine (durene diamine) and 6FDA, was chosen as the polymer matrix. Microporous PI particles were introduced into 6FDA-Durene matrix to prepare “all polyimide” mixed-matrix membranes. It is expected that the “all polyimide” chemical structure of the fillers and the matrix would induce excellent interfacial compatibility between them, and thus the microporous PI particles may improve the gas separation performance of MMMs even at a lower filler loading level. Thus, the gas permeability and selectivity performance of the MMMs were investigated. Furthermore, the anti-plasticization ability for CO_2_ of MMMs was also studied.

## 2. Materials and Methods

### 2.1. Materials

Tris(4-nitrophenyl)amine (AR, 97%), hydrazine monohydrate (AR, 99%), palladium (10% on carbon; Pd/C), 4′-nitroacetophenone (AR, 98%), thionyl chloride (AR, 98%), 4-aminobenzonitrile (AR, 98%), trifluoromethanesulfonic acid (AR, 98%), 2,3,5,6-tetramethyl-1,4-phenylenediamine (Durene, AR, 98%;), isoquinoline (AR, 97%), and pyridine (AR, 99%) were purchased from TCI Chemical Co., Ltd (Shanghai, China). and used without purification. Pyromellitic dianhydride (PMDA, AR, 97%) and 4,4’-(hexafluoroisopropylidene)-diphthalic andydride (6FDA, AR, 97%) were purchased from Shanghai Aladdin Bio-Chem Technology Co., Ltd. and used after vacuum sublimation. *N*-methylpyrrolidone (NMP, AR, 99%), 1,3,5-trimethylbenzene (Mesitylene, AR, 99%), and *N,N*-dimethylformamide (DMF, AR, 99%) were purchased from Energy Chemical Co., Ltd. and used after vacuum distillation. The other analytical grade reagents or solvents were purchased from Energy Chemical Co., Ltd (Shanghai, China). and used as received.

### 2.2. Synthesis and Membranes Fabrication

#### 2.2.1. Synthesis of Tris(4-aminophenyl)amine (TAPA)

Catalytic amount of palladium was added into a solution of tris(4-nitrophenyl)amine (3.80 g, 10 mmol) in ethanol (75 mL) in a three-neck flask. Then, hydrazine monohydrate (10 mL, 206 mmol) was added dropwise into the flask within 5 h under stirring. Afterwards, the mixture was refluxed under argon atmosphere for 24 h. After cooling down to room temperature and filtration to remove the catalyst, the crude product was recrystallized with ethanol to obtain white needle crystal. Yield: 70%. 

^1^H NMR (500 MHz, DMSO-*d6*, *δ*, ppm): 6.62–6.58 (d, 6H), 6.46–6.42 (d, 6H) and 4.70 (s, 6H). ^13^C NMR (500 MHz, DMSO-*d6*, *δ*, ppm): 143.6, 139.1, 124.5 and 115.2. Anal. calcd for C_18_H_18_N_4_: C (74.46), H (6.25) and N (19.30), found: C (74.64), H (6.21) and N (19.27). MS (EI, *m/z*): [M] + calcd for C_18_H_18_N_4_: 290.36. Found: 290.09.

#### 2.2.2. Synthesis of 1,3,5-Tris(4-nitrophenyl)benzene (TNPB)

Thionyl chloride (12 mL, 165 mmol) was added dropwise into the mixture of 4′-nitroacetophenone (16.52 g, 100 mmol) and ethanol (50 mL) in a three-neck flask within 1 h under stirring. Then, the mixture was refluxed under argon atmosphere for additional 4 h. After neutralized with deionized water, the yellow product was washed with ethyl ether and ethanol. Yield: 54%. 

^1^H NMR (500 MHz, DMSO-*d6*, *δ*, ppm): 8.38–8.25 (m, 6H), 8.05–8.01 (d, 6H) and 7.50 (s, 3H). Anal. calcd for C_24_H_15_N_3_O_6_: C (65.31), H (3.43) and N (9.52), found: C (64.65), H (3.50) and N (9.51). MS (EI, *m/z*): [M]+ calcd for C_24_H_15_N_3_O_6_: 441.10. Found: 441.00.

#### 2.2.3. Synthesis of 1,3,5-Tris(4-aminophenyl)benzene (TAPB)

Similar to the synthetic procedure of TAPA, precursor TNPB was used instead of tris(4-nitrophenyl)amine to synthesize TAPB. White needle crystal was obtained. Yield: 68%. 

^1^H NMR (500 MHz, DMSO-*d6*, *δ*, ppm): 7.51–7.47 (m, 9H), 6.70–6.66 (d, 6H) and 5.21 (s, 6H). ^13^C NMR (500 MHz, DMSO-*d6*, *δ*, ppm): 148.8, 142.1, 128.5, 127.9, 120.8 and 114.7. Anal. calcd for C_24_H_21_N_3_: C (82.02), H (6.02) and N (11.96), found: C (81.30), H (5.58) and N (11.88). MS (EI, *m/z*): [M]+ calcd for C_24_H_21_N_3_: 351.17. Found: 351.00.

#### 2.2.4. Synthesis of 2,4,6-Tris(4-aminophenyl)-1,3,5-triazine (TAPT)

Trifluoromethanesulfonic acid (4 mL, 44 mmol) was added dropwise into 4-Aminobenzonitrile (1.54 g, 13 mmol) under ice bath within 5 h under vigorous stirring. The system was kept stirring under argon atmosphere for 12 h. After the reaction completed, water was employed to quench the reaction and neutralize. The crude product was washed with ethyl ether and ethanol. Faint yellow powders were obtained. Yield: 84%.

^1^H NMR (500 MHz, DMSO-*d6*, *δ*, ppm): 8.36–8.34 (d, 6H), 6.71–6.69 (d, 6H) and 5.91 (s, 6H). ^13^C NMR (500 MHz, DMSO-*d6*, *δ*, ppm): 170.0, 153.4, 130.6, 123.4 and 113.6. Anal. calcd for C_21_H_18_N_6_: C (71.17), H (5.12) and N (23.71), found: C (69.19), H (4.99) and N (22.51). MS (EI, *m/z*): [M]+ calcd for C_21_H_18_N_6_: 354.16. Found: 354.00.

#### 2.2.5. Synthesis of Microporous Polyimides

Highly crosslinked microporous polyimide networks (AP, BP, and TP) were synthesized by one-pot polycondensation of trifunctional amines monomer (TAPA, TAPB, and TAPT) with PMDA [24,25,26].

TAPA (0.58 g, 2 mmol) and PMDA (0.66 g, 3 mmol) were dissolved in 10 mL mixed solvent of NMP and mesitylene (vol:vol = 1:1) before catalytic amount of isoquinoline was added. Then, polymerization was carried out in the heating schedule: 60 °C for 2 h, 90 °C for 2 h, 120 °C for 2 h, 150 °C for 2 h, 180 °C for 2 h, and 195 °C for 4 h under vigorous stirring. After the polycondensation completed, the suspension was poured into methanol and filtered. The precipitate was extracted with methanol and dried at 120 °C under vacuum. Brown powder AP was obtained.

The polymerizations of BP and TP were carried out in similar procedure of AP; precursors TAPB and TAPT were used instead of TAPA. Yellow or dark yellow powders were obtained for BP or TP.

#### 2.2.6. Fabrication of Pristine Membrane (6FDA-Durene)

Durene diamine (16.4 g, 100 mmol) was dissolved in 350 mL of DMF. Then, 6FDA (44.4 g, 100 mmol) was added into the solution. The mixture was stirred at 0 °C for 2 h and 30 °C for additional 8 h to obtain viscous polyamic acid (PAA) solution. After that, pyridine (3 mL) and acetic anhydride (38 mL) were used as catalyst and dehydrating agent and added into the PAA solution for 8 h to obtain polyimide. After the polycondensation was completed, the solution was poured into methanol and filtered. The precipitate was extracted with methanol and dried at 120 °C under vacuum. White fiber was obtained.

The 15 wt.% solid content 6FDA-Durene in DMF solution was prepared and cast on a dust free glass plate. Solvent evaporation under vacuum at 250 °C to obtain the freestanding membrane.

#### 2.2.7. Fabrication of MMMs 

Taking the MMMs with AP as an example, the filler content is expressed as:

(1) $$\phi =\frac{m_{\mathrm{AP}}}{m_{\mathrm{AP}}+m_{6\mathrm{FDPI}}}$$

The MMM that contains 5 wt.% AP was named as 5AP/6FDA-Durene. The other MMMs were named accordingly. Only the preparation of 5AP/6FDA-Durene is described here as an example.

AP (0.1 g) was dispersed in DMF (11 mL). Then, 6FDA-Durene (1.9 g) was dissolved in the suspension. The mixture was cast on a dust free glass plate. Solvent evaporation under vacuum at 250 ℃ was performed to obtain the freestanding membrane.

### 2.3. Material Characterization Methods

Nuclear magnetic resonance (NMR) was recorded on a Bruker AVANCE 500 MHz NMR spectrometer. Triamine monomers, which were dissolved in deuterated dimethyl sulfoxide (DMSO-*d6*) with tetramethylsilane (TMS) before the test, were analyzed by ^1^H NMR and ^13^C NMR spectra. Microporous PI particles were analyzed by ^13^C cross-polarization with magic angle spinning (CP/MAS) spectra. Mass spectra was recorded on a Thermofisher DSQ II EI mass spectrometer. Elemental analysis was recorded on a Elementar Vario EL cube elemental analyzer. Fourier-transform infrared spectra (FTIR) was recorded on a Bruker TENSOR 27 FTIR spectrometer. Microporous PI particles were analyzed by KBr transmittance method and the films were analyzed by attenuated total refection (ATR) method. The 77 K N_2_ adsorption–desorption isotherms of microporous PI particles were recorded on a micrometrics ASAP 2020 absorption analyzer. The Brunner−Emmet−Teller (BET) specific surface area of samples can be calculated automatically by micrometrics ASAP 2020 according to the N_2_ adsorption capacity at *p*/*p*_0_= 0.07, 0.08, 0.12, 0.16, and 0.20. According to the classical Langmuir model, gas molecules form monolayer adsorption on the surface of porous materials. The specific surface area (*a_s_*) of porous materials can be obtained by the saturated adsorption capacity of monolayer gas (*n*_m_) and the size of gas molecules. For example, the cross-sectional area (*a*_m_) of a nitrogen molecule is 0.162 nm^2^ at 77 K. The specific surface area of porous material can be described by:

*a_s_ = a_m_·L·n_m_* = 9.76 × 10*^4^·n_m_* (2)

When van der Waals force also exists between gas molecules, gas molecules will form multi-molecular layer adsorption, where the saturated adsorption capacity (*n*_a_) is greater than that of the monolayer adsorption capacity (*n*_m_). The monolayer adsorption capacity can be described by

(3) nm=na·(1−pp0)

The specific surface area is calculated by Equation (3) after the monolayer adsorption capacity is obtained, in which specific surface area is named Brunner−Emmet−Teller (BET) specific surface area. All samples were treated at 150 °C for 6 h before testing. The mean value of BET specific surface areas was tested three times.

Pore size distributions were analyzed by nonlocal density functional theory (NLDFT) method. Dynamic light scattering (DLS) was recorded on a Malvern MasterSizer 2000 particle size analyzer. The mean value of particle sizes was tested three times. Thermal gravimetric analysis (TGA) was recorded on a Shimadzu TGA-50H thermogravimetric analyzer. Measurements were performed in the range of 50–800 °C at a heating rate of 10 °C·min^−1^ under N_2_ atmosphere. Differential scanning calorimetry (DSC) was recorded on a NET ZSCH DSC 204 thermal analyzer. Measurements were performed in the range of 50–400 °C at a heating rate of 10 °C·min^−^^1^ under N_2_ atmosphere. Dynamic thermomechanical analysis (DMA) was recorded on TA Q800 DMA analyzer. Measurements were performed in the range of 50–500 °C at a heating rate of 10 °C·min^−1^ under N_2_ atmosphere. Mechanical properties of MMMs were recorded on CMT6103 tensile machine. Wide-angle X-ray diffraction (XRD) was recorded on a Rigaku SmartLab X-ray diffractometer. Measurements were performed in the range of 5–60° at the scanning speed of 5°·min^−1^. Instrument used CuK_α_ radiation (λ = 1.54 Å). Field-emission scanning electron microscope (SEM) was recorded on Hitachi S-4800. The films were fractured at 77 K and sputtered with chromium electron conducting layer in vacuum before testing. High-resolution transmission electron microscope (TEM) was recorded on JEOL JEM-2010HR. Measurements were performed on 200 kV acceleration voltage. Skeletal densities of microporous PIs were recorded on a Micrometrics Accupyc Helium pycnometry. Density of films were recorded on a Mettler Toledo ML204. Measurements were performed by buoyancy method. The FFV of microporous PIs and films were calculated using Equation (4):

(4) $$FFV=\frac{V-1.3\cdot V_{w}}{V},$$

where *V* is the molecular weight of the repeat unit of polymer and *V_w_* is the van del Waals volume estimated using Bondi’s method [27,28]. For MMM, *V_w_* was estimated as [29]:

(5) $$V_{w}=m_{d}\cdot V_{wd\;}+m_{c}\cdot V_{wc},$$

where *m_c_* and *m_d_* are the mole fraction of continuous and dispersed phase, respectively. *V_wc_* and *V_wd_* are the van del Waals volume of continuous and dispersed phase, respectively.

### 2.4. Gas Transport Characterization Methods

Single component ideal gas permeation tests were performed on a variable pressure constant-volume apparatus (Lanmo Tech. Co., Ltd, Changsha, China). Measurements were performed at 30 °C and 100 psi. Gas permeability coefficient were calculated follow:

(6) $$P=\frac{273\times 10^{10}}{760}\times \frac{V\cdot l\cdot \frac{dp}{dt}}{A\cdot T\cdot p_{0}\cdot \frac{76}{14.7}},$$

where *P* is the gas permeability coefficient (Barrer, 1 Barrer = 10^−10^ cm^3^ (STP) cm·cm^−2^·s^−1^·cm·Hg^−1^); *V* is the volume of the downstream (cm^3^); *A* is the effective area of membrane (cm^2^); *l* is the membrane thickness (cm); *p* is the pressure on downstream (kPa); and *t* is the time (s). Diffusivity coefficients (*D*, cm^2^·s^−1^) were calculated following the time-lag method:

(7) $$D=\frac{l^{2}}{6\cdot \theta },$$

where *θ* is the time-lag (s). Thus, the solubility coefficient (*S*, cm^3^(STP)·cm^−3^·cmHg^·−1^) as calculated as *P* = *D·S*. The ideal selectivity for two gas were calculated as:

(8) αxy=PxPy,

We used Arrhenius equation to gain activation energy of permeation (*E_p_*, kJ·mol^−1^):

(9) $$P=P_{0}\cdot exp\left(\frac{-E_{P}}{R\cdot T}\right),$$

where *P*_0_ is the permeability coefficient in the standard state (273 K and 1 bar); *R* is the molar gas constant (8.314 J·mol^−1^·K^−1^); and *T* is absolute temperature (K). The temperature-dependence test was conducted from 25 to 50 ℃ at 100 psi.

CO_2_ and CH_4_ adsorption–desorption isotherms of microporous PI particles and MMMs were recorded on a BEL-SORB high pressure sorption instrument. Measurements were performed in the range 0–1000 kPa at 30 °C. The solubility coefficients (*S*, cm^3^(STP) ·cm^−3^·cmHg^−1^) and dual-mode sorption were fitted to [19]:

(10) $$S=\frac{C}{p}=k_{D}+\frac{C_{H}^{\prime }\cdot b}{1+b\cdot p^{\prime }}$$

where *C* is the adsorption quantity at equilibrium (cm^3^(STP)·g^−1^); *p* is the equilibrium pressure (kPa); *k_D_* is Henry’s law constant (cm^3^·g^−1^·kPa^−1^); *b* is affinity coefficient (kPa^−1^); and $C_{H}^{\prime }$ is Langmuir coefficient (cm^3^·g^−1^).

Microcavity of films were confirmed by positron annihilation lifetime spectroscopy (PALS): Neglecting of para-positronium self-annihilation (*τ*_1_) and free annihilation (*τ*_2_), the target ortho-positronium (*o*-Ps, *τ*_3_) were obtained. The cavity size was calculated by using the Tao–Eldrup Equation [30]:

(11) $$\tau_{3}^{-1}=2\cdot \left[1-\frac{r}{r_{0}}+\frac{1}{2\cdot \pi }\cdot \sin\left(\frac{2\cdot \pi \cdot r}{r_{0}}\right)\right],$$

where *τ*_3_ is the lifetime of *o*-Ps (ns); *r* is the radius of the pores in film; and *r*_0_ = *r* + 1.66 (Å). Assuming the pore to be spherical, the free volume can be obtained by the Equation:

(12) $$V=\frac{4}{3}\cdot \pi \cdot r^{3},$$

The fractional free volume was calculated by the semi-empirical Equation:

(13) $$FFV=0.0018\times \left(V_{3}\cdot I_{3}\right),$$

where *I*_3_ is *o*-Ps intensity related to the number of micropores.

## 3. Results and Discussion

### 3.1. Synthetic Procedure and Structures of Monomers, Microporous PIs and 6FDA-Durene 

**Scheme 1:**
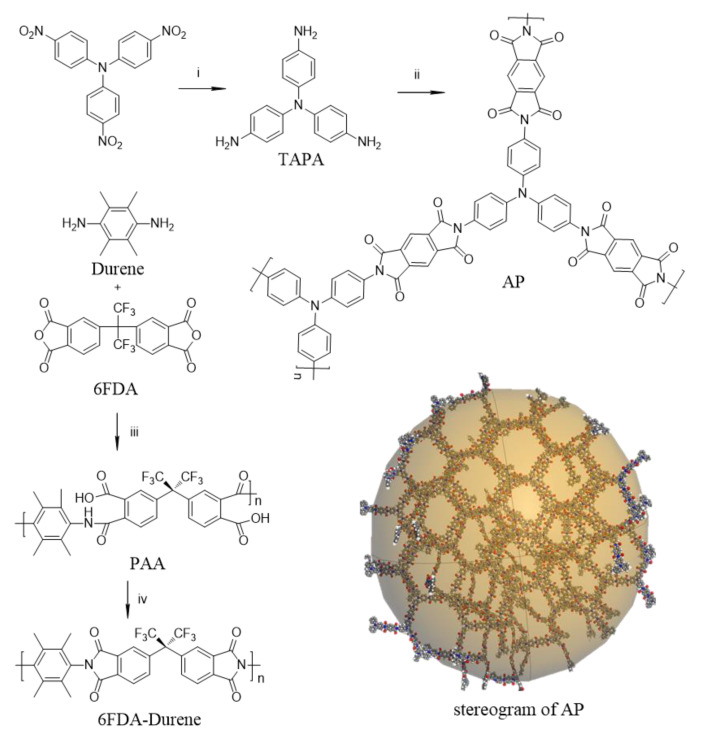
Synthetic procedure and structures of TAPA, AP, and 6FDA-Durene. Reagents and conditions: (**i**) H_2_N-NH_2_·H_2_O, P_d_/C and ethanol reflux for 24 h; (**ii**) PMDA, isoquinoline, NMP and mesitylene mix solvent, heating schedule; (**iii**) DMF, reaction in ice bath for 8 h; and (**iv**) pyridine and acetic anhydride for 8 h. The picture inserted in the lower right corner shows the 3D structure of AP micropores.

Three microporous PI particles (AP, BP, and TP), were prepared by the reaction of tris(4-aminophenyl)amine (TAPA), 1,3,5-tris(4-aminophenyl)benzene (TAPB), and 2,4,6-tris(4-aminophenyl)-1,3,5-triazine (TAPT) with pyromellitic dianhydride (PMDA), respectively. The structures of TAPA, AP, and 6FDA-Durene are illustrated in Scheme 1. Tris(4-nitrophenyl)amine was reduced by hydrazine hydrate to form triamine TAPA. One-pot polycondensation of TAPA and PMDA were performed to obtain microporous particle AP. Two-step polycondensation of 6FDA and Durene were performed to obtain the polymer matrix 6FDA-Durene. The structures and synthesis routes of TAPB, TAPT, BP, and TP are illustrated in Schemes S1 and S2. The results of mass spectra, elemental analysis, ^1^H NMR, and ^13^C NMR certificated that the monomers were prepared successfully. The details are described in the Experimental Section 2.2.

The microporous PI particles (AP, BP, and TP) are all insoluble in any solvent. Therefore, solid-state ^13^C CP/MAS NMR (Figures S1–S3 and Table S1 in supplementary material) and FTIR spectra (Figure S4 in supplementary material) were used to confirm that they were synthesized successfully [24,25,26]. 

ATR-FTIR was used to characterize the pristine membrane and the “all polyimide” MMMs (Figure S5 in supplementary material). The complete imidization was confirmed by the absence of peaks at 1620–1680 and 3300 cm^−1^, which are associated with the stretching of carbonyl group (C=O) on PAA and the stretching of N–H on amine monomer, respectively. Most transmission bands of “all polyimide” MMMs, such as symmetric and asymmetric stretching vibration of C=O (1725 and 1775 cm^−1^), stretching vibration of C–N on the five-membered imide ring (1355 cm^−1^), and stretching vibration of C–F on 6FDA structural unit (1142 cm^−1^), coincide with 6FDA-Durene [31,32].

### 3.2. Morphology of the Microporous PIs

**Figure 1:**
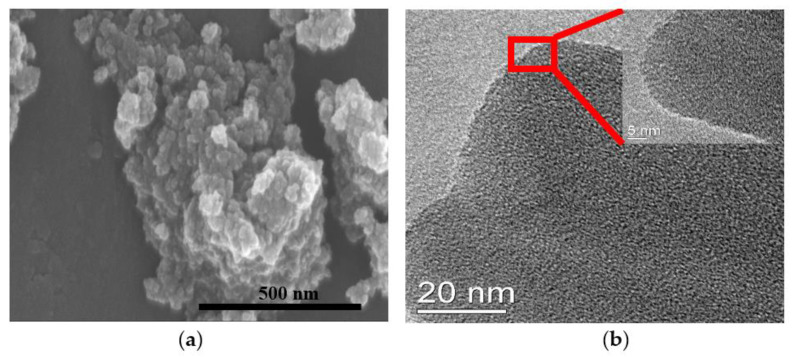
(**a**) SEM; and (**b**) high-resolution TEM of AP.

Figure 1a and Figure S6b,c (in supplementary material) show that the microporous particles AP, BP, and TP displayed coral-like agglomerations composed of tiny particles [25]. The size of aggregated particles gained by SEM is approximately 500 nm, smaller than the volume weighted mean particle size (2.62–3.14 μm) measured by DLS (Table 1). This may be due to the serious aggregation feature of microporous PIs in ethanol, which is a non-solvent for microporous PIs and used as mobile phase for DLS measurement. 

High-resolution TEM of AP and its amplification are shown in Figure 1b. The white region represents micropores and the black region represents polyimide backbone [33]. We can see the interpenetrating network architecture in microporous PI corresponding to the image inserted in Scheme 1, which may allow fast penetration of gas [24].

### 3.3. Porosity Structures of the Microporous PIs and 6FDA-Durene

**Figure 2:**
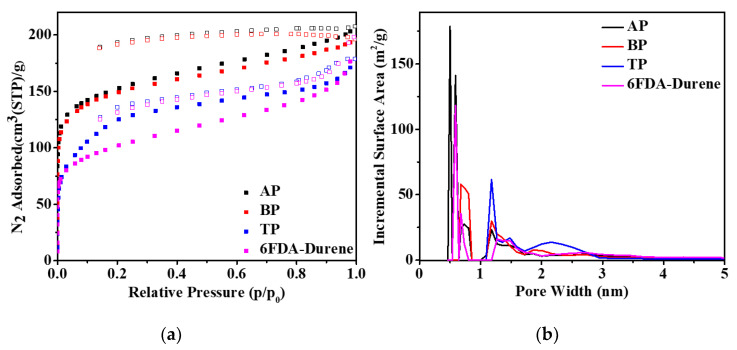
(**a**) N_2_ adsorption (solid) and desorption (hollow) isotherms of AP, BP, TP, and 6FDA-Durene at 77 K; and (**b**) pore size distribution calculated by NLDFT.

N_2_ adsorption isotherm of microporous PIs and 6FDA-Durene were tested at 77 K to explain the porosity. Type Ⅳ adsorption isotherm and obvious hysteresis curves are shown in Figure 2a for all samples [24]. Rapid N_2_ uptake in low pressure region (*p*/*p*_0_ < 0.1) was caused by large amount of micropores in the samples. The results confirm that microporous PI and 6FDA-Durene belong to MOP. The BET specific surface area of AP, BP, TP, and 6FDA-Durene are 520.58, 510.52, 453.46, and 353.87 m^2^·g^−1^ respectively. (Table 1). Microporous adsorption saturation appeared at the point of *p*/*p*_0_ = 0.1. After that, mesoporous adsorption took place. It can be seen from the hysteresis curve that the micropores of TP and 6FDA-Durene are constructed by parallel plate-like packing particles. However, AP and BP display ink-bottle-shaped micropores [34]. NLDFT pore size distribution data (Figure 2b) confirmed that microporous PIs had a large number of micropores less than 2 nm. Especially, AP has more ultra-micropores of 0.5–0.7 nm compared with BP, TP, and 6FDA-Durene, which show a typical hierarchical porous structure, and may be beneficial to ensure fast penetration of gas while maintaining its selectivity [35]. 

### 3.4. Interfacial Compatibility Studies of the “All Polyimide” MMMs

As is known, in the preparation of composite films, the difference of density usually leads to the settlement of high-density dispersion components, which seriously affects the homogeneity of material properties. Thus, the density of microporous PIs and matrix 6FDA-Durene was characterized, and the results are shown in Table 2. The density of 6FDA-Durene (1.33 g·cm^−3^) is close to those of AP, BP, and TP (1.29, 1.36, and 1.41 g·cm^−3^, respectively), indicating good processing stability when combined them together. The measured densities of the MMMs by buoyancy method are in the range of 1.31–1.34 g·cm^−3^. The density of the MMMs is also predicted by maxwell equation:

*ρ_MMM_ = ρ_c_Φ_c_ + ρ_d_Φ_d_*, (14)

where *ρ*_c_ and *ρ*_d_ are densities of continuous phase and dispersed phase and *Φ*_c_ and *Φ*_d_ are volume fractions of continuous phase and dispersed phase [36]. The results show that the measured densities of the MMMs are approximate to the predicted values, which implies the good compatibility between the microporous PIs and the matrix in the composites films.

**Figure 3:**
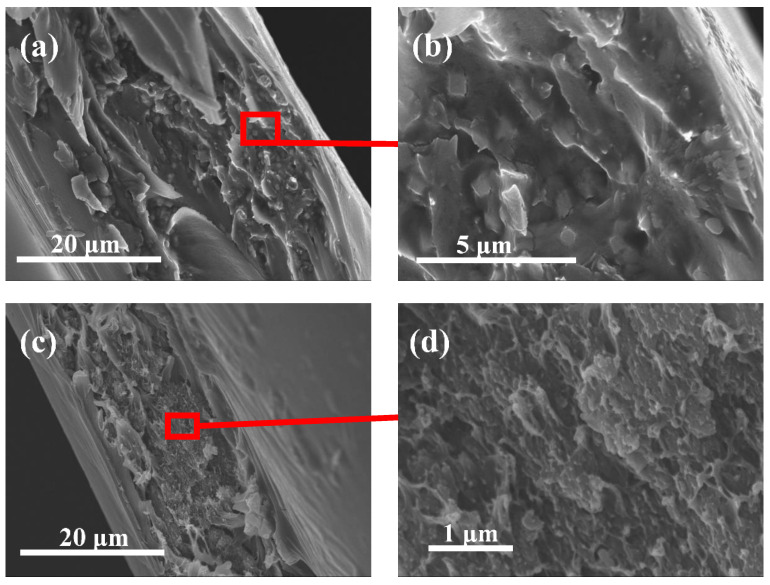
Cross-section SEM images of MMM with AP loading: (**a,b**) 5AP/FDA-Durene and amplification; and (**c,d**) 10AP/FDA-Durene and amplification.

The cross-section SEM images of the MMMs can show the interfacial compatibility of the composites more directly. As shown in Figure 3, the “all polyimide” MMMs with the thickness of about 30 μm are dense homogeneous membranes. The “all polyimide” MMMs belong to dense homogeneous membranes, whose thicknesses are around 30 μm. The dispersed phase AP is uniformly distributed into the continuous phase 6FDA-Durene with very good polymer–particle interfacial adhesion. Interfacial defects were hardly found in the MMMs even at AP loading up to 10 wt.%, which is beneficial to maintain the good selectivity of the composite films [37]. This is really not common in traditional MMMs with micron-scale fillers. MMMs of 5BP/6FDA-Durene and 5TP/6FDA-Durene have the same phenomenon with 5AP/6FDA-Durene, as shown in Figure S7 (in supplementary material).

XRD patterns of AP, BP, TP, pristine membrane, and “all polyimide” MMMs are shown in Figure S8 (in supplementary material). Broad diffraction peaks confirm the amorphous nature of the microporous PIs, pristine membrane, and the MMMs. *d*-Spacing was obtained by Bragg’s equation:

2·*d*·sin*θ* = n·*λ*, (15)

The diffraction peaks at 22.3° corresponds to *d*-spacing of 4.09 Å for AP (Table 2), which can be assigned to main interchain distance and microporous size. The amorphous halo match with the micropore size distribution in NLDFT and stereogram of the interpenetrating network architecture. The peaks located at 13.6° and 25.3° of the membranes are assigned to the amorphous domain and π–π stacking, respectively [38]. After the incorporation of AP, the maximum amorphous diffraction peaks of the “all polyimide” MMMs shifted toward lower angle, and the average *d*-spacing of 6FDA-Durene, 5AP/6FDA-Durene, and 10AP/6FDA-Durene are 6.57, 6.34, and 6.30, respectively, implying that the molecular chain segments of 6FDA-Durene stacked more tightly after loading of AP. 

### 3.5. Thermal and Mechanical Performances of Microporous PIs and Films

**Figure 4:**
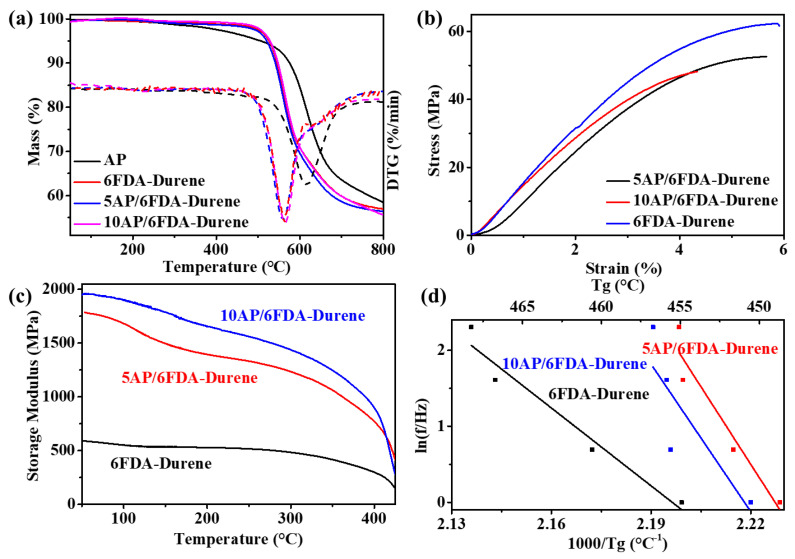
(**a**) TGA (solid line) and DTG (dotted line) of AP and the MMMs with AP loading; (**b**) stress–strain of MMMs with AP loading; (**c**) storage Modulus of MMMs with AP loading; and (**d**) Arrhenius plots of MMMs with AP loading.

TGA test indicated that the dispersed phase and continuous phase of the “all polyimide” MMMs had good thermal stability (Figure 4a and Figure S9 in supplementary material). The 5 wt.% decomposition temperatures of 6FDA-Durene, 5AP/6FDA-Durene, 10AP/6FDA-Durene, 5BP/6FDA-Durene, and 5TP/6FDA-Durene are 529, 523, 529, 529, and 532 °C, respectively. Especially the microporous PIs (AP, BP, and TP) have a maximum weight loss rate (DTG) at 616, 629, and 640 °C, respectively. The thermal stability of AP, BP, and TP are much higher than those of common MOF materials [39]. Thus, the thermal stability of the designed “all polyimide” MMMs should be prior to that of the MOF-based MMMs, which is very important for practical applications of MMMs. The small amount of weight loss of AP, BP, and TP in the range of 100–500 °C is thermal desorption of the gases or moisture in micropores, which is common in MOPs [40].

As shown in Figure 4b, Figure S10 (in supplementary material), and Table 2, the addition of microporous PIs into 6FDA-Durene can enhance the Young’s modulus of the composite films but decrease the tensile strength and elongation at break of the MMMs slightly. The increase of Young’s modulus of the MMMs indicates the compatibility of the PIs fillers and the PI matrix. However, the rigid nature of the microporous PI particles leads to the embrittlement of the MMMs and the decrease of tensile strength and elongation at break. 

Loss tangent (tan*δ*) and storage modulus (*E’*) of 6FDA-Durene, 5AP/6FDA-Durene, and 10AP/6FDA-Durene at four frequencies (1, 2, 5, and 10 Hz) were recorded by DMA (Figure S11 in supplementary material). The slopes of Arrhenius plots (Figure 4d) were used to gain the activation energies (*E*_α_) of glass transition (*α*-relaxation) [41]. *T*_g_ of MMMs with AP loading has little change compared with 6FDA-Durene. The activation energy of *a*-relaxation and *E’* can effectively characterize the stiffness of the chain. Table 3 display that *E*_α_ is 284, 537 and 566 kJ·mol^−1^ for 6FDA-Durene, 5AP/6FDA-Durene, and 10AP/6FDA-Durene, respectively. More energy is required for the motion of molecular segment in MMMs compared to 6FDA-Durene. The *E’* value of 10AP/6FDA-Durene reaches up to approximate 2 GPa at room temperature due to the chain segment stiffness effect reported by Koros [12].

### 3.6. Gas Transport Property

**Figure 5:**
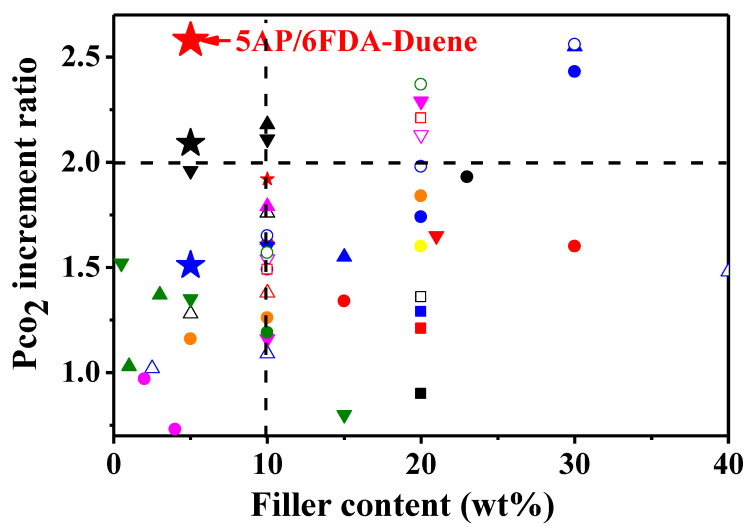
$P_{CO_{2}}$ increment ratio of PI based MMMs with MOPs or MOFs loading compared to pristine PI membrane. (★), (★), and (★) are 6FDA-Durene with 5% of AP, BP, and TP loading (this work), respectively. Triangles are 6FDA-Durene with NH_2_-MIL-101 (▲) [45], CNT-MIL (△) [45], HKUST-1 (▲) [20], HKUST-IL (△) [20], UiO-66-NH_2_ (▲) [21], PSM-MOF (△) [21], ZIF-71 (▲) [46], PgC5Cu (▲) [47], [Cu(6L)]^2+^@13X (▼) [42], Ni(dobdc) (▼) [48], ZIF-71 (▼) [49], ZIF-67 (▼) [50], ZIF-67-IL2 (▽) [50], and Zeolite T (▼) [51] loading. Circles are 6FDAM with mmen-Mg_2_(dobpdc) (●) [52], PPN (●) [53], HKUST-1 (●) [39], branch HKUST-1 (○) [39], ns-CuBDC (●) [54], 2D-COF (●) [55], 3D-COF (○) [55], ZMOF (●) [56], and NH_2_-MIL-53(Al) nano particle (●) [57] loading. (□) [23], (□) [23], and (□) [23] are Matrimid with PPI-1, PPI-2 and PPI-3 loading, respectively. (■) [23], (■) [23], and (■) [23] are 6FDA-IMM with PPI-1, PPI-2, and PPI-3 loading, respectively.

As shown in Table 4, compared with 6FDA-Durene, the $P_{CO_{2}}$ of “all polyimide” MMMs were significantly improved. $P_{CO_{2}}$ increment ratio of the “all polyimide” MMMs in this work and other PI based MMMs compared with their pristine membrane versus filling content is illustrated in Figure 5; the details are shown in Table S2 (in supplementary material). The $P_{CO_{2}}$ increment ratio of 5AP/6FDA-Durene is very significant among the existing PI-based MMMs with filler content below 20 wt.%. Diffusivity coefficient was measured by time-lag. Table 4 further shows that the increase of diffusivity coefficient leads to the increase of permeability coefficient. As shown in Table 1, AP has the highest FFV and porosity among the three microporous PIs particles, which is beneficial to improve the diffusivity coefficient of the “all polyimide” MMMs [42]. The skeleton of AP form an interpenetrating network architecture, which might form a high-rate gas transport channel [15,43]. Besides, the AP skeleton contains hierarchical micropores ranging from 0.5 to 2 nm (Figure 2b), in which the micropores with the dimension of beyond 6.6 Å (twice larger than CO_2_ dynamic diameter) should ensure fast penetration of gas [24,44]. Moreover, excellent interfacial compatibility between AP and 6FDA-Durene may be beneficial to the effect utilization of porosity and free volume of AP fillers during gas transport. As a result, 5AP/6FDA-Durene shows the highest $P_{CO_{2}}$ increment ratio compared with most MMMs with MOFs content below 20 wt.%. However, the ideal selectivity of the “all polyimide” MMMs shows a slight decrease but almost maintains at a same level compared with the pristine membrane due to the good compatibility.

As shown in Table 4, the $P_{CO_{2}}$ and CO_2_ solubility coefficient ($S_{CO_{2}}$) of 5AP/6FDA-Durene (1291.13 Barrer and 27.45 × 10**^−^**^2^ cm^3^(STP)·cm**^−^**^3^·cmHg**^−^**^1^ respectively) are much higher than those of 10AP/6FDA-Durene (959.16 Barrer and 16.35 × 10**^−^**^2^ cm^3^(STP)·cm**^−^**^3^·cmHg**^−^**^1^, respectively). From the dynamic mechanical properties of the composite films (Figure 4c), one can see that the molecule chain segments of MMMs become more rigid as the AP content increases, which was verified by the *α*-relaxation activation energy (Table 3). As a result, the micropores of fillers on the interface were blocked, which led to the reduction of CO_2_ sorption and solution on 10AP/6FDA-Durene [50,57].

**Figure 6:**
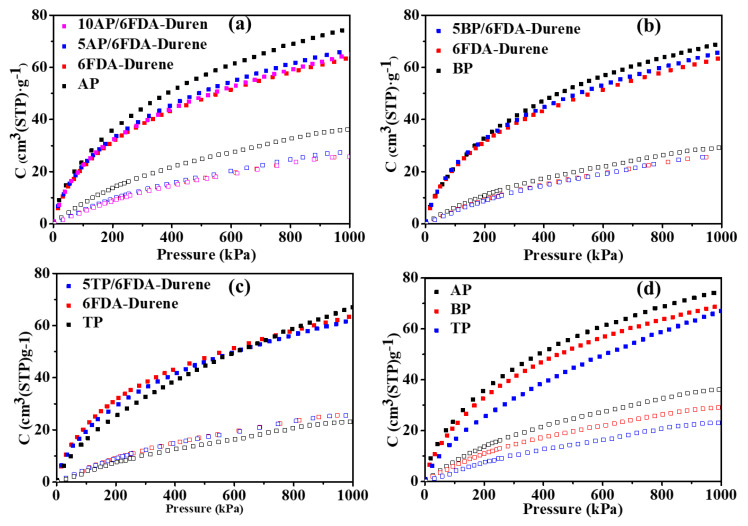
CO_2_ (**solid square**) and CH_4_ (**hollow square**) uptake at 30 °C of: (**a**) MMMs with AP loading; (**b**) MMMs with BP loading; (**c**) MMMs with TP loading; and (**d**) microporous PIs.

To further investigate the gas transport properties of the “all polyimide” MMMs, high pressure sorption experiments were performed, and the results are shown in Figure 6. The order of CO_2_ uptake properties at 30 °C of AP, BP, and TP is AP > BP > TP (Figure 6d), which is consistent with the BET results of the three particles. Among the three microporous PIs, CO_2_ uptake of AP and BP exceed 6FDA-Durene matrix due to their higher porosity. As a result, the quantity adsorbed and solubility coefficient of CO_2_ in 5AP/6FDA-Durene and 5BP/6FDA-Durene are slightly higher than those in 6FDA-Durene at 30 °C and 100 psi. The CO_2_ diffusion coefficient ($D_{CO_{2}}$) deduced from $S_{CO_{2}}$ also prove that diffusion behavior dominated the permeation [21] (Table 5).

To investigate the sorption behavior in MMMs, dual-mode sorption was used to fit the CO_2_ isotherms. The results are shown in Table 5. The Henry’s law constant (*k_D_*) represents the gas dissolution behavior in the dense region of the film. The Langmuir coefficient ($C_{H}^{\prime }$) and affinity coefficient (*b*) represent the gas dissolution behavior in the microporous region of the film [45]. $C_{H}^{\prime }$ value of 5AP/6FDA-Durene is 48.69 cm^3^·g^−1^, which is higher than that of 6FDA-Durene (44.02 cm^3^·g^−1^). A higher Langmuir coefficient represents a larger number of micro-cavities and FFV in 5AP/6FDA-Durene, resulting in the boost in diffusivity coefficient [58]. This is the main reason the quantity adsorbed of CO_2_ in 5AP/6FDA-Durene is higher than that in 6FDA-Durene. However, 6FDA-Durene has higher CO_2_ affinity coefficient than 5AP/6FDA-Durene. This is because the −CF_3_ group on 6FDA dianhydride monomer unit [42] can lead to CO_2_ dipole polarization. $C_{H}^{\prime }$ value of 10AP/6FDA-Durene decreased to 43.88 cm^3^·g^−1^, which demonstrated the blockage of the micropores in the dispersed phase.

**Figure 7:**
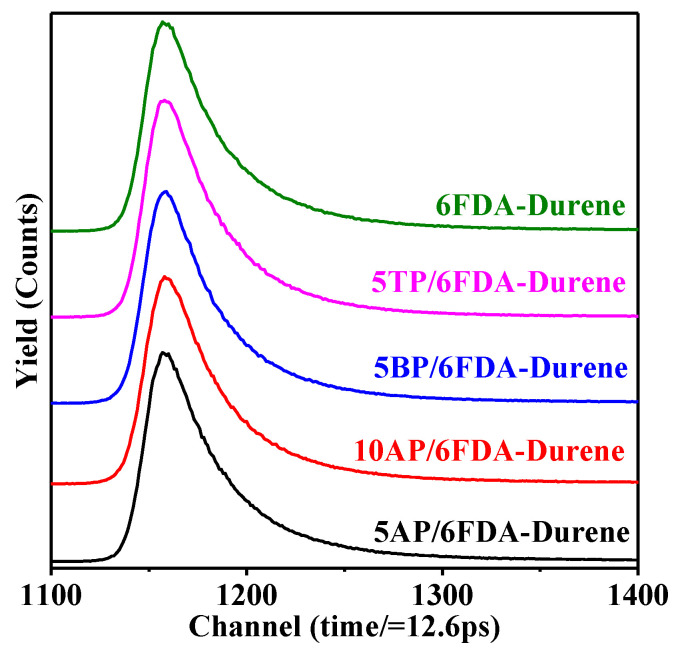
PALS of 6FDA-Durene and “all polyimide” MMMs.

Positron annihilation technique was used to studies the FFV in the MMMs. The results are shown in Figure 7 and Table 5. 5AP/6FDA-Durene not only has the highest FFV value (4.9%) in this work, but also has larger cavity volume (*V*_3,_ 245.7 Å^3^) and more micropores (*I*_3_, 11.1%) than 6FDA-Durene (*V*_3_ = 244.6 Å^3^, and *I*_3_ = 10.7%) [59]. Both the size and the number of micro-cavities are improved, which confirmed that the introduction of AP with typical hierarchical porous structure can increase the FFV in MMMs [60]. This is the reason 5AP/6FDA-Durene has the highest $P_{CO_{2}}$ in our work. With the increase of AP content, *I*_3_ continued to increase to 11.4%, while FFV and *V_3_* decreased in 10AP/6FDA-Durene (FFV = 4.8%, and *V_3_* = 234.7 Å^3^) compared with 5AP/6FDA-Durene. It showed that the number of micro-cavities increased with the increase of AP content, but the blockage of the pores in dispersed phase of 10AP/6FDA-Durene decreased the pore volume. The FFV determined by group contribution method is different from that of PALS because of different models and assumptions [61], but the variation tendency is almost the same.

**Figure 8:**
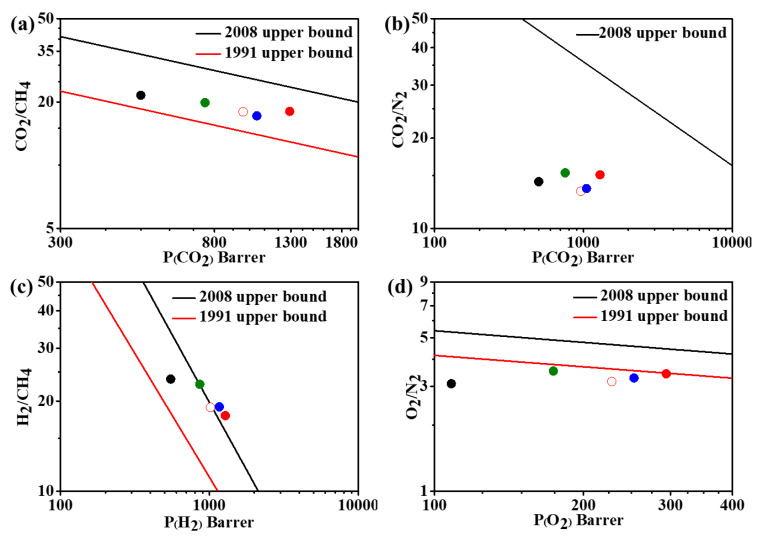
Robeson upper bound limit of (**a**) CO_2_/CH_4_ separation; (**b**) CO_2_/N_2_ separation; (**c**) H_2_/CH_4_ separation; and (**d**) O_2_/N_2_ separation. (●), (●), (**○**), (●), and (●) are 6FDA-Durene, 5AP/6FDA-Durene, 10AP/6FDA-Durene, 5BP/6FDA-Durene, and 5TP/6FDA-Durene, respectively.

It is useful to further modify the gas separation performance of 6FDA-Durene because its CO_2_/CH_4_ separation performance exceeds the 1991 Robeson upper bound. As shown in Figure 8, after incorporating microporous PIs into matrix, 5AP/6FDA-Durene, 5BP/6FDA-Durene, and 5TP/6FDA-Durene can improve $P_{CO_{2}}$ significantly, which makes the CO_2_/CH_4_ separation performance close to the 2008 upper bound limit (Figure 8a). CO_2_/N_2_ (Figure 8b), O_2_/N_2_ (Figure 8c), and H_2_/CH_4_ (Figure 8d) separation performance were also improved. Especially, the performance of 5AP/6FDA-Durene and 5BP/6FDA-Durene in H_2_/CH_4_ separation surpasses the 2008 upper bound limit. The ratio of the initial slopes of the CO_2_ and CH_4_ adsorption isotherm in Henry’s law region was used to calculated the Henry adsorption selectivity [62] (Figure S12 and Table S3 in supplementary material). Selectivity of CO_2_/CH_4_ for AP was 4.02. According to the estimation of Maxwell’s equations, with the increase of filler content, the gas separation performance of the composite membrane will change from the matrix to the filler. The CO_2_ permeability coefficient of AP is assumed to be very large. The slightly decrease of CO_2_/CH_4_ selectivity of “all polyimide” MMMs compared to 6FDA-Durene is fitted for the estimation of Maxwell’s equations. It can explain that, although AP has many ultra-fine micropores less than 1 nm in NLDFT pore size distribution (Figure 2b), MMMs with AP loading do not display molecular sieving performance for gas molecules with smaller dynamic diameter [54]. Despite the low Henry adsorption selectivity of microporous PIs, the membrane separation selectivity is not greatly lost, in fact, due to the good two-phase compatibility in “all polyimide” MMMs. In addition, the increase of CO_2_ permeation of 5AP/6FDA-Durene after modification is fitted to the estimation of Maxwell’s equations [21,39]. The blockage of the pores in dispersed phase of 10AP/6FDA-Durene makes it deviate from Maxwell’s equation model. Therefore, low filling content (5 wt.%) can significantly increase gas permeation in “all polyimide” MMMs.

**Figure 9:**
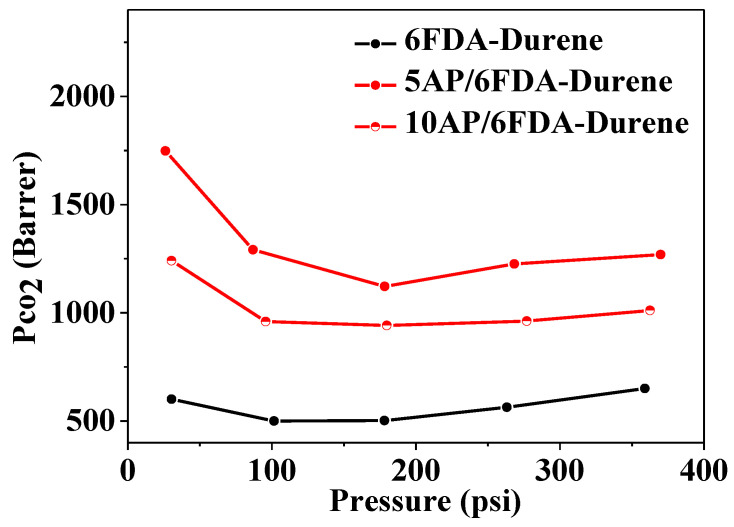
Plasticizing resistance of MMMs with AP loading.

It is well known that we should avoid CO_2_ plasticization during CO_2_/CH_4_ separation. Dual-mode sorption and plasticization both affect the CO_2_/CH_4_ separation performance of the membrane. 6FDA-Durene displays a typical curve in pressure swing gas permeability test in Figure 9, which was seriously affected by dual-mode sorption and plasticization [21,48]. First, as $S_{CO_{2}}$ decreases with the increase of pressure, $P_{CO_{2}}$ decreases before 100 psi. After that, plasticization is dominant, and $P_{CO_{2}}$ increases. After incorporation of AP, especially in the case of 10AP/6FDA-Durene, the MMMs shows a CO_2_ plasticization pressure of 300 psi, indicating the excellent plasticizing resistance. This is because the interfacial chain segment becomes stiff, which was contributed by the compatibility between filler and matrix.

With the increase of temperature, the transport rate of gas molecules increases, resulting in the improvement of the permeability coefficient [63]. However, in our work, the permeability coefficient of CO_2_ decreases with the rise of temperature (Figure S13a), and the activation energy (*E*_p_) of CO_2_ permeation is negative [64] (Table S4). This is likely because the solubility coefficient of CO_2_ decreases with the rise of temperature. In addition, the permeability coefficient of N_2_ and CH_4_ has little change with temperature. Therefore, low temperature is beneficial for the “all polyimide” MMMs to separate CO_2_/CH_4_ (Figure S13b).

## 4. Conclusions

In summary, three kinds of microporous PI particles (AP, BP, and TP) were prepared by a one-pot polycondensation reaction of three triamine monomers (TAPA, TAPB, and TAPT) with a dianhydride monomer PMDA, respectively. The as-prepared PI particles show a typical inter-penetrating crosslinked network and hierarchical porous structure. Among them, AP has the highest BET specific surface area of 520.58 m^2^·g^−1^ and FFV value of 18% and a micropores size ranging from 5 to 20 Å (twice beyond CO_2_ dynamic diameter). A series of “all polyimide” mixed-matrix membranes was obtained by introducing the PI particles into an intrinsic microporous polyimide matrix 6FDA-Durene. The SEM images and maxwell density mode verified that the PI particles are homogenously dispersed into the PI matrix with high interfacial compatibility. Compared with the pristine membrane, permeability coefficients of N_2_, O_2_, H_2_, CH_4_ and CO_2_ of the “all polyimide” MMMs increase significantly. Especially the $P_{CO_{2}}$ of 5AP/6FDA-Durene reaches up to 1291.13 Barrer, which is 2.58 times that of the pristine 6FDA-Durene membrane. The $P_{CO_{2}}$ increment ratio in 5AP/6FDA-Durene is state-of-the-art in the existing PI-based MMMs with filler content below 20 wt.%, and the H_2_/CH_4_ separation performance surpass the 2008 Robeson upper limit after modification. Meanwhile, compared with pristine membrane, the chain segment between the dispersed phase and the continuous phase bind together and become stiff in 10AP/6FDA-Durene, in which tensile modulus rose from 1 to 1.66 GPa and activation energy of *α*-relaxation rose from 284 to 566 kJ·mol^−1^, resulting in excellent plasticizing resistance in CO_2_ separation beyond 300 psi. This behavior is different from the previously reported MMMs in which high filler content was required to achieve a high permeability increase that would generally lead to the significant agglomeration or phase separation of MMMs. It is believed that the excellent interfacial miscibility between the PI fillers and the PI matrix induce the effect utilization of porosity and free volume of AP fillers during gas transport. Thus, a higher diffusion coefficient of MMMs was observed than that of the pristine PI membrane. However, due to the low adsorption selectivity of CO_2_/CH_4_ of microporous polyimide, the ideal selectivity of the “all polyimide” MMMs shows a slight decrease but almost maintains at the same level compared with the pristine membrane due to the good compatibility. These “all polyimide” MMMs with high compatibility are promising gas separation membranes due to their high gas permeability. Searching for high adsorption selectivity fillers for the enhancement of the gas separation performance in MMMs is the focus of future work.

## Figures and Tables

**Table 1:** Porosity property, particle size, and FFV of AP, BP, TP, and 6FDA-Durene.

	*S*_BET_ (m^2^·g^−1^)	Particle Size (μm) ^a^	*D* 0.9 (μm) ^b^	FFV (%) ^c^
AP	520.58 ± 27.89	3.14 ± 0.06	5.62	18.00
BP	510.52 ± 30.50	2.98 ± 0.08	7.23	11.56
TP	453.46 ± 16.47	2.62 ± 0.03	6.50	NA
6FDA-Durene	353.87 ± 6.28	NA	NA	14.93

^a^ Determined by DLS, volume weighted mean distribution; ^b^ determined by DLS, <90%; ^c^ obtained by group contribution method which contributed by Bondi.

**Table 2:** Physical properties of continuous phase and dispersed phase.

	*d*-Spacing (Å) ^a^	Young’s Modulus (GPa)	Elongation at Break (%)	Tensile Strength (MPa)	Density (g·cm^−3^) ^b^	Density (g·cm^−3^)
AP	4.09	NA	NA	NA	NA	1.29 ^c^
BP	4.07	NA	NA	NA	NA	1.36 ^c^
TP	4.12	NA	NA	NA	NA	1.41 ^c^
6FDA-Durene	6.57	1.00 ± 0.26	5.92 ± 1.53	53.89 ± 1.77	NA	1.33 ^d^
5AP/FDA-Durene	6.34	1.59 ± 0.01	4.35 ± 1.11	46.89 ± 2.43	1.32	1.31 ^d^
10AP/FDA-Durene	6.30	1.66 ± 0.02	3.29 ± 0.59	48.95 ± 5.75	1.32	1.31 ^d^
5BP/FDA-Durene	6.69	1.52 ± 0.01	5.05 ± 0.78	49.50 ± 2.06	1.33	1.32 ^d^
5TP/FDA-Durene	6.72	1.47 ± 0.22	3.94 ± 0.57	45.53 ± 6.69	1.33	1.34 ^d^

^a^ Obtained by Bragg’s equation; ^b^ theoretical calculation results based on densities of dispersed phase and continuous phase: *ρ*_MMM_ = *ρ*_c·_*Φ*_c_ + *ρ*_d·_*Φ*_d_; ^c^ obtained by helium replacement method; ^d^ obtained by buoyancy method.

**Table 3:** Storage modulus (*E*’) and activation energies (*E*_α_).

	*E*’ (GPa) ^a^	*E*_α_ (kJ·mol^−1^)
6FDA-Durene	0.60 ± 0.01	284
5AP/FDA-Durene	1.79 ± 0.11	537
10AP/FDA-Durene	1.95 ± 0.09	566

^a^ Measurement at 1 Hz and 50 °C.

**Table 4:** Gas permeation and ideal selectivity of pristine membrane and “all polyimide” MMMs.

		H_2_	N_2_	O_2_	CH_4_	CO_2_	H_2_/CH_2_	O_2_/N_2_	CO_2_/N_2_	CO_2_/CH_4_
6FDA-Durene	P ^a^	549.14	34.95	108.11	23.20	500.17	23.67	3.09	14.31	21.56
	D ^b^	43.35	23.41	28.63	10.11	21.38	4.28	1.22	0.91	2.12
	S ^c^	12.67	1.49	3.78	2.30	23.39	5.52	2.53	15.67	10.19
5AP/FDA-Durene	P	1277.25	85.58	293.91	71.45	1291.13	17.88	3.43	15.09	18.07
	D	73.68	54.74	60.60	26.41	47.03	2.79	1.11	0.85	1.78
	S	17.33	1.56	4.85	2.71	27.45	6.41	3.10	17.56	10.14
10AP/FDA-Durene	P	1015.20	72.13	228.14	53.32	959.16	19.04	3.16	13.30	17.99
	D	125.48	76.24	97.95	27.38	58.65	4.58	1.28	0.77	2.14
	S	8.09	0.95	2.33	1.95	16.35	4.15	2.46	17.29	8.40
5BP/FDA-Durene	P	1164.25	77.15	253.00	60.89	1047.43	19.12	3.28	13.58	17.20
	D	116.44	59.86	86.31	28.13	56.74	4.14	1.44	0.95	2.02
	S	10.00	1.29	2.93	2.16	18.46	4.62	2.27	14.32	8.53
5TP/FDA-Durene	P	861.58	49.20	173.83	37.89	753.07	22.74	3.53	15.31	19.88
	D	100.71	35.29	63.57	16.53	35.58	6.09	1.80	1.01	2.15
	S	8.55	1.39	2.73	2.29	21.17	3.73	1.96	15.18	9.24

^a^ Permeability coefficient, unit: Barrer, 1 Barrer = 10^−10^ cm^3^ (STP) cm·cm^−2^·s^−^^1^·cmHg^−^^1^; ^b^ diffusivity coefficient was measured by time-lag method, unit: 10^−8^ cm^2^·s^−1^. S was deduced based on P = D·S; ^c^ solubilities coefficient, unit: 10^−2^ cm^3^(STP) ·cm^−3^·cmHg^−1^.

**Table 5:** CO_2_ dual-mode sorption fitting parameters at 30 °C and FFV.

	${S_{CO_{2}}}^{a}$	${D_{CO_{2}}}^{b}$	*k_D_* ^c^	${C_{H}^{\prime }}^{d}$	*b* ^e^	R^2^	*I* (%)	*τ*_3_ (ns)	*V*_3_ (Å^3^)	FFV ^f^ (%)	FFV ^g^ (%)
6FDA-Durene	13.80	36.25	0.025	44.02	0.0077	99.96	10.7	3.4	244.6	4.7	14.93
5AP/FDA-Durene	14.60	88.44	0.025	48.69	0.0069	99.93	11.1	3.4	245.7	4.9	15.78
10AP/FDA-Durene	13.93	68.87	0.026	43.88	0.0082	99.97	11.4	3.3	234.7	4.8	15.99
5BP/FDA-Durene	14.26	73.48	0.026	45.65	0.0076	99.98	10.6	3.4	246.9	4.7	15.21
5TP/FDA-Durene	13.81	54.54	0.025	43.04	0.0067	99.92	6.7	3.0	202.4	2.4	NA

^a^ Solubility coefficient was measured by high pressure sorption, unit: 10^−2^ cm^3^ (STP) ·cm^−3^·cmHg^−^^1^; D was deduced based on P = D·S; ^b^ diffusivity coefficient, unit: 10^−8^ cm^2^·s^−1^; ^c^ Henry’s law constant, unit: cm^3^·g^−1^·kPa^−1^; ^d^ Langmuir coefficient, unit: cm^3^·g^−1^; ^e^ affinity coefficient, unit: 10^−3^ kPa^−1^; ^f^ determined by PALS; ^g^ obtained by group contribution method which contributed by Bondi.

## Data Availability

The data presented in this study are available upon request from the corresponding author.

## Supplementary Materials

The following are available online at https://www.mdpi.com/article/10.3390/polym13081329/s1, Scheme S1: Synthetic procedure and structures of TAPB and BP; Scheme S2: Synthetic procedure and structures of TAPT and TP; Figure S1: ^13^C CP/MAS NMR spectra of AP; Figure S2: ^13^C CP/MAS NMR spectra of BP; Figure S3: ^13^C CP/MAS NMR spectra of TP; Figure S4: FTIR spectra of AP, BP, and TP; Figure S5: ATR spectra of 6FDPPI and “all PI” MMMs; Figure S6: DLS of AP, BP, and TP and SEM of BP and TP; Figure S7: Cross-section SEM images of 5BP/6FDA-Durene and 5TP/6FDA-Durene; Figure S8: XRD patterns of microporous PIs, 6FDA-Durene, and “all polyimide” MMMs; Figure S9: TGA of BP, TP 5BP/6FDA-Durene, and 5TP/6FDA-Durene; Figure S10: Stress–strain of 5BP/6FDA-Durene and 5TP/6FDA-Durene; Figure S11: DMA of 6FDA-Durene, 5AP/6FDA-Durene, and 10AP/6FDA-Durene; Figure S12: Henry fit for idea adsorption solution theory (IAST) of AP, BP, and TP; Figure S13. Gas permeability and ideal selectivity of 5AP/6FDA-Durene in different temperature; Table S1: Assignments for Figures S1–S3; Table S2: The ratio of $P_{CO_{2}}$ of PI base MMMs to pristine PI membranes; Table S3: Henry’s law region fit for IAST of microporous PIs; Table S4: 5AP/6FDA-Durene activation energy (E_p_) of permeation for CO_2_, N_2_ and CH_4_.
