# Supplementary material for: “All Polyimide” Mixed Matrix Membranes for High Performance Gas Separation
Source: Polymers (Basel). 2021 Apr 19;13(8):1329. doi: 10.3390/polym13081329 (PMC8073420; doi:10.3390/polym13081329)
Supplement: Supplementary file 1 [file polymers-13-01329-s001.zip › polymers-1172427-supplementary.pdf]

Supporting Information

"All Polyimide" Mixed Matrix Membranes for High Performance Gas Separation

Li Maijun[1], Zheng Zhibo[1*], Zhang zhiguang[2], Li Nanwen[2*], Liu Siwei[2], Chi Zhenguo[1], Xu Jiarui[1], and Zhang Yi[1*]

†PCFM Lab, GD HPPC Lab, Guangdong Engineering Technology Research Centre for High-performance Organic and Polymer Photoelectric Functional Films, State Key Laboratory of Optoelectronic Materials and Technologies, School of Chemistry, Sun Yat-sen University, Guangzhou 510275, China.

‡State Key Laboratory of Coal Conversion, Institute of Coal Chemistry, Chinese Academy of Sciences. Taiyuan, China

Scheme S1. Synthetic procedure and structures of TAPB and BP. 2

Scheme S2. Synthetic procedure and structures of TAPT and TP. 3

Figure S1. ^{13}C CP/MAS NMR spectra of AP. 4

Figure S2. ^{13}C CP/MAS NMR spectra of BP. 4

Figure S3. ^{13}C CP/MAS NMR spectra of TP. 5

Figure S4. FTIR spectra of AP, BP and TP. 5

Figure S5. ATR-FTIR spectra of 6FDA-Durene and "all polyimide" MMMs. 6

Figure S6. DLS of AP, BP, TP and SEM of BP, TP 7

Figure S7. Cross-section SEM images of 5BP/6FDA-Durene and 5TP/6FDA-Durene 7

Figure S8. XRD patterns of microporous PIs, 6FDA-Durene and "all polyimide" MMMs. 8

Figure S9. TGA of BP, TP, 5BP/6FDA-Durene and 5TP/6FDA-Durene. 8

Figure S10. Stress-strain of 5BP/6FDA-Durene, 5TP/6FDA-Durene and 6FDA-Durene 9

Figure S11. DMA of 6FDA-Durene, 5AP/6FDA-Durene and 10AP/6FDA-Durene at 1 Hz, 2 Hz, 5 Hz, 10 Hz respectively 9

Figure S12. Henry fit for idea adsorption solution theory (IAST) of AP, BP and TP 10

Figure S13. Gas permeability and ideal selectivity of 5AP/6FDA-Durene in different temperature... 10

Table S1. Assignments for **Figure. S1-S3** 10

Table S2. The ratio of P_{CO_2} of PI base MMMs to pristine PI membranes 10

Table S3. Henry's law region fit for IAST of microporous PIs. 15

Table S4. 5AP/6FDA-Durene activation energy (E_P) of permeation for CO_2, N_2 and CH_4 15

REFERENCES 15

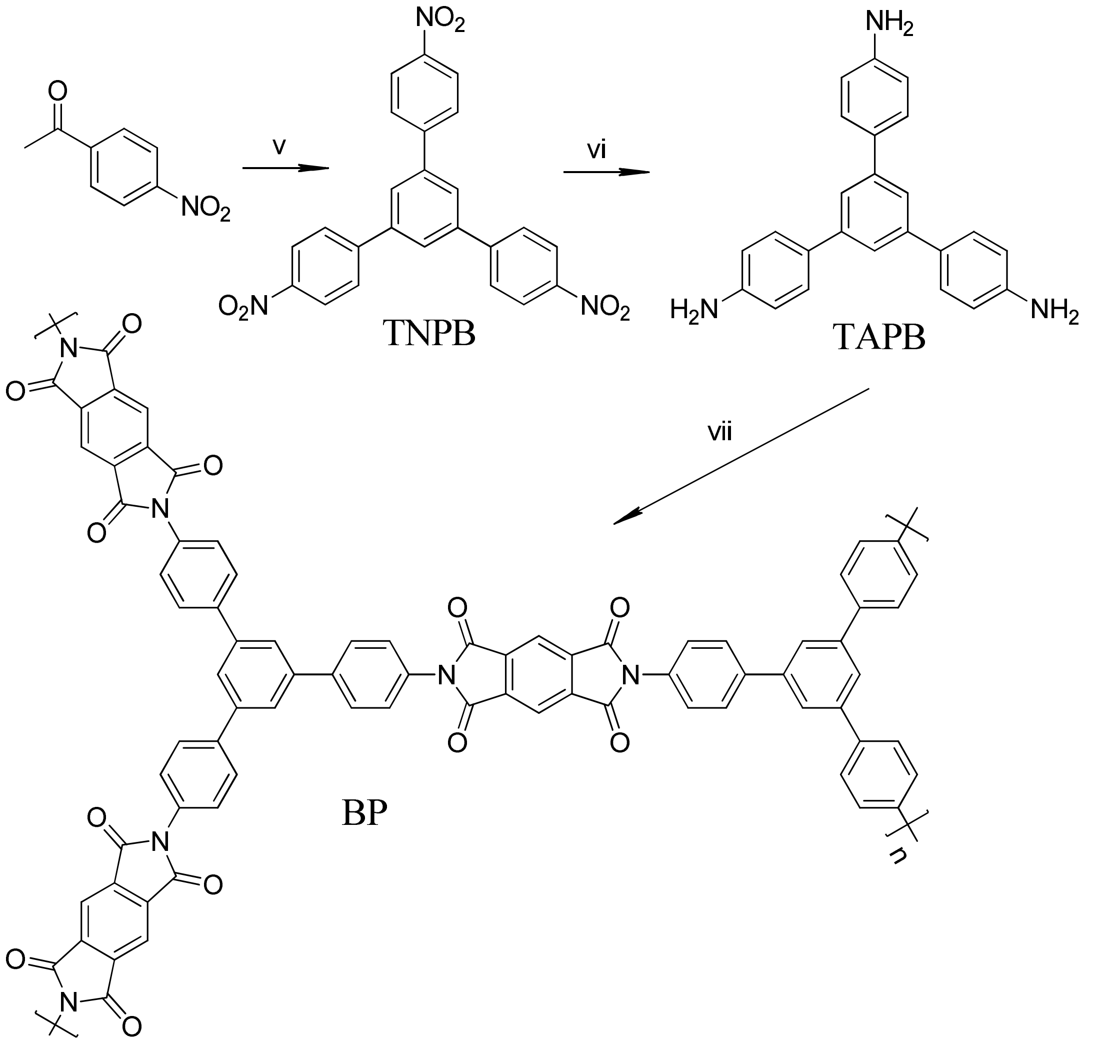

Scheme S1. Synthetic procedure and structures of TAPB and BP. Reagents and conditions: (v) thionyl chloride and ethanol reflux for 4 h; (vi) $H_2N\text{-}NH_2{\cdot}H_2O$, Pd/C and ethanol reflux for 24 h; (vii) isoquinoline, NMP and mesitylene mix solvent, heating schedule.

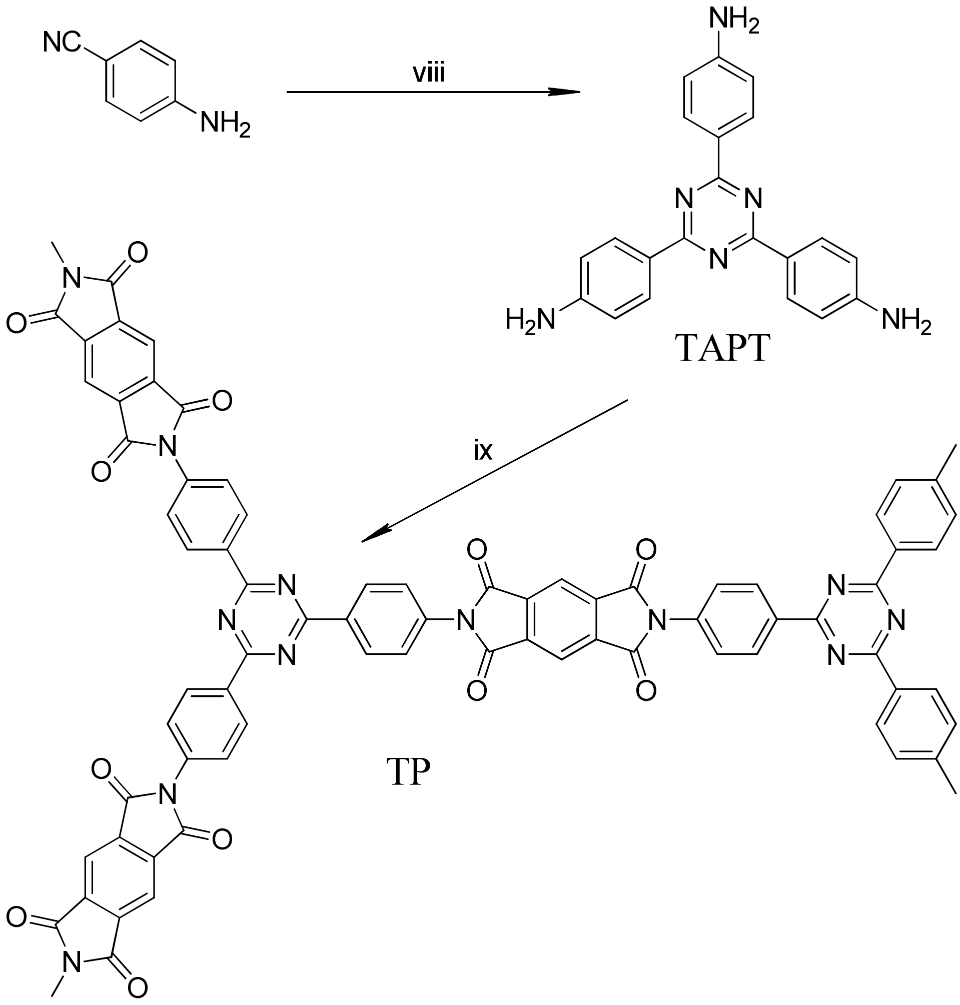

Scheme S2. Synthetic procedure and structures of TAPT and TP. Reagents and conditions: (viii) trifluoromethanesulfonic acid and 4-aminobenzonitrile reaction in ice bath for 12 h; (ix) isoquinoline, NMP and mesitylene mix solvent, heating schedule.

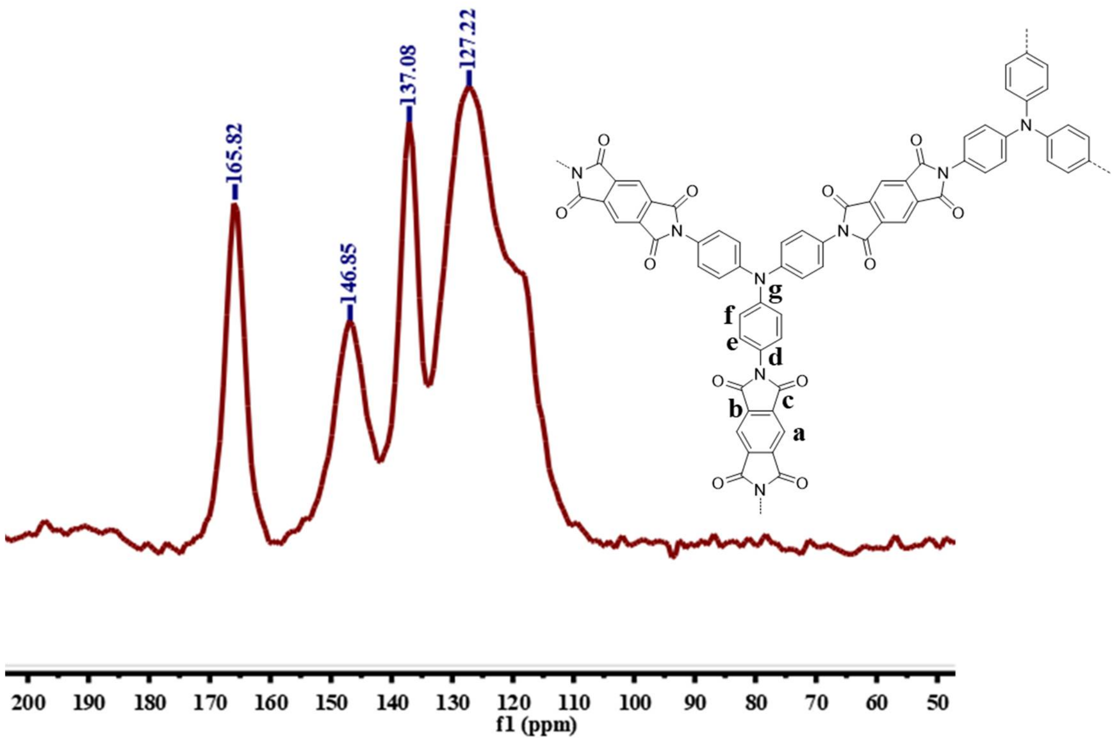

Figure S1. ^{13}C CP/MAS NMR spectra of AP.

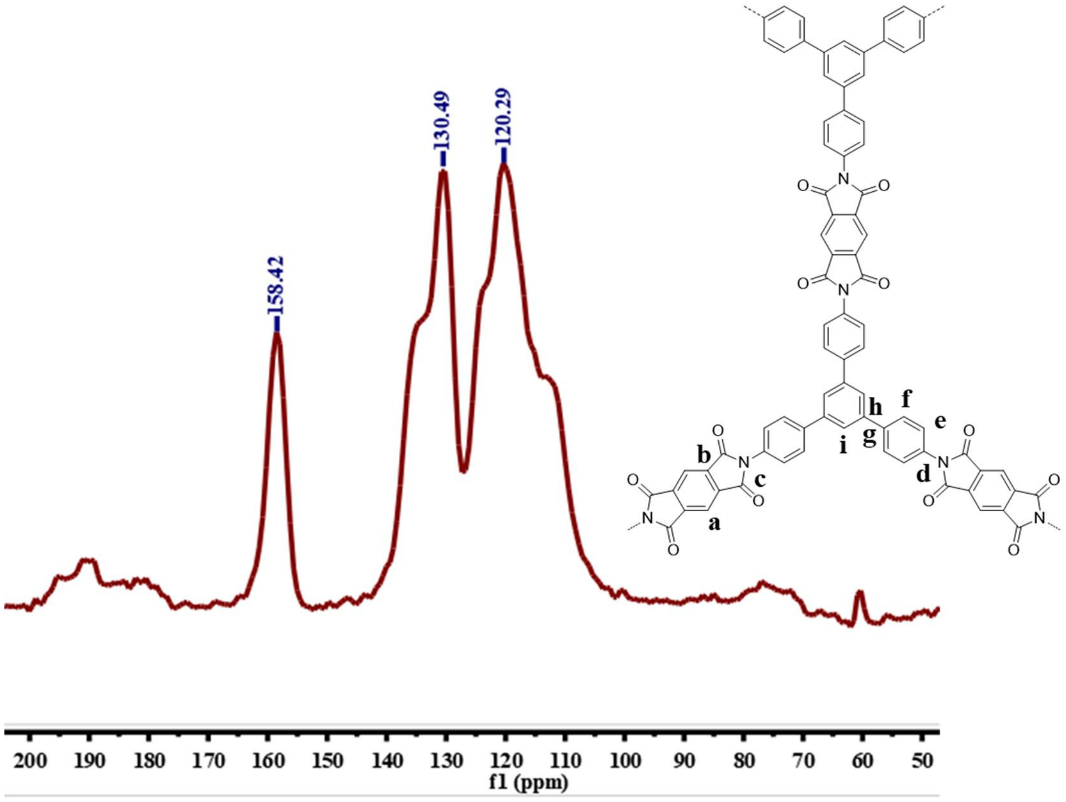

Figure S2. ^{13}C CP/MAS NMR spectra of BP.

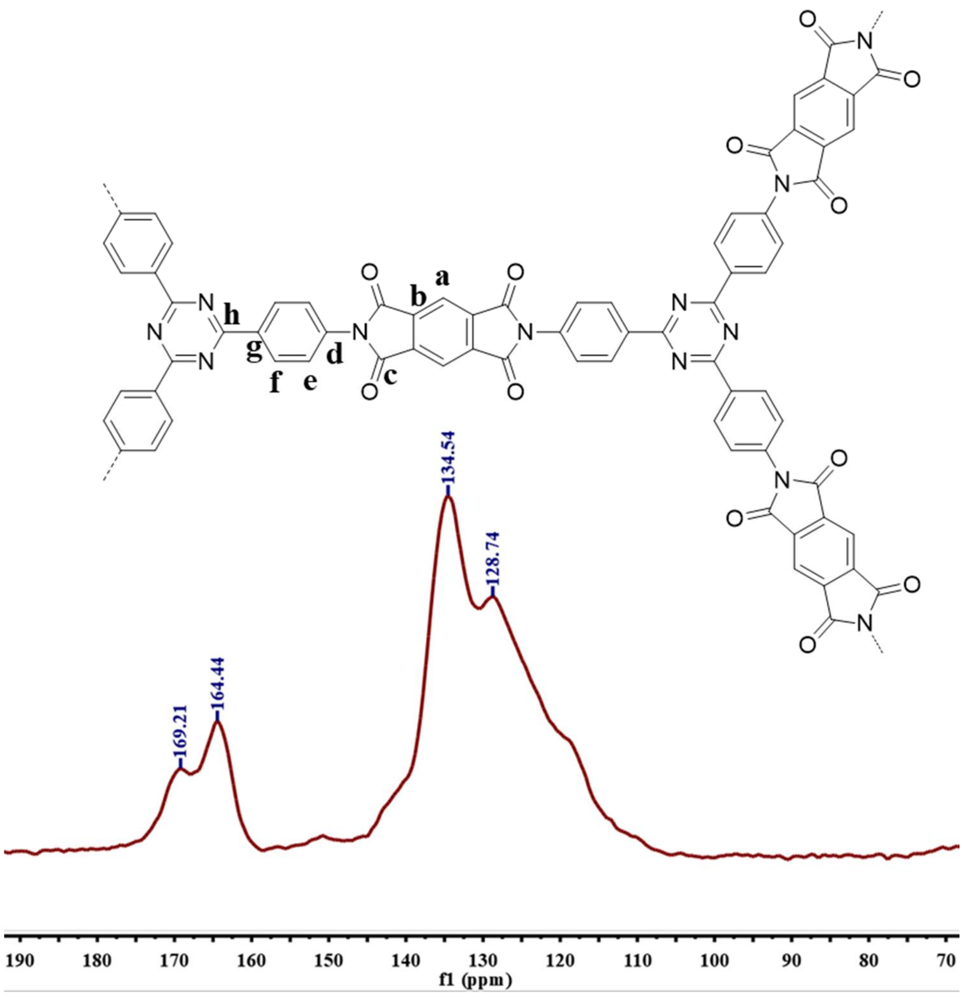

Figure S3. ^{13}C CP/MAS NMR spectra of TP.

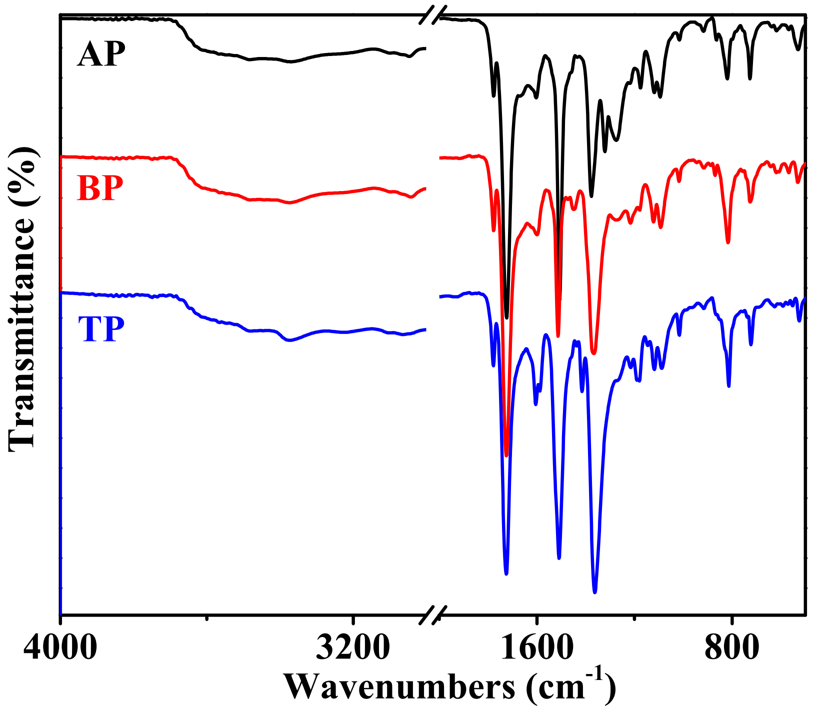

Figure S4. FTIR spectra of AP, BP and TP.

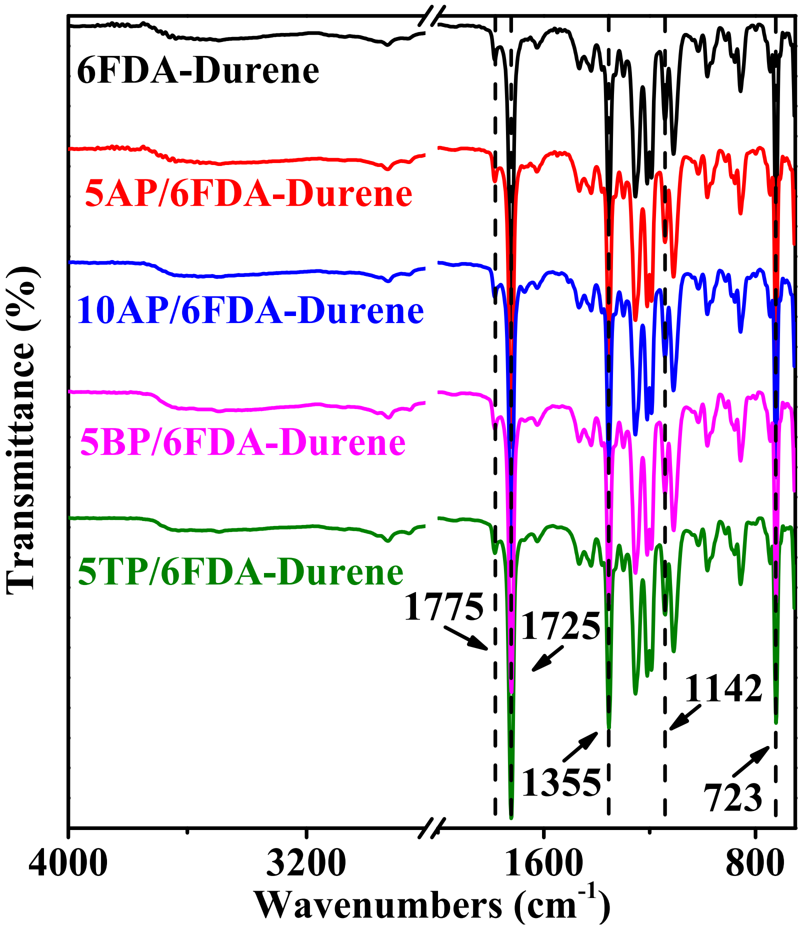

Figure S5. ATR-FTIR spectra of 6FDA-Durene and "all polyimide" MMMs.

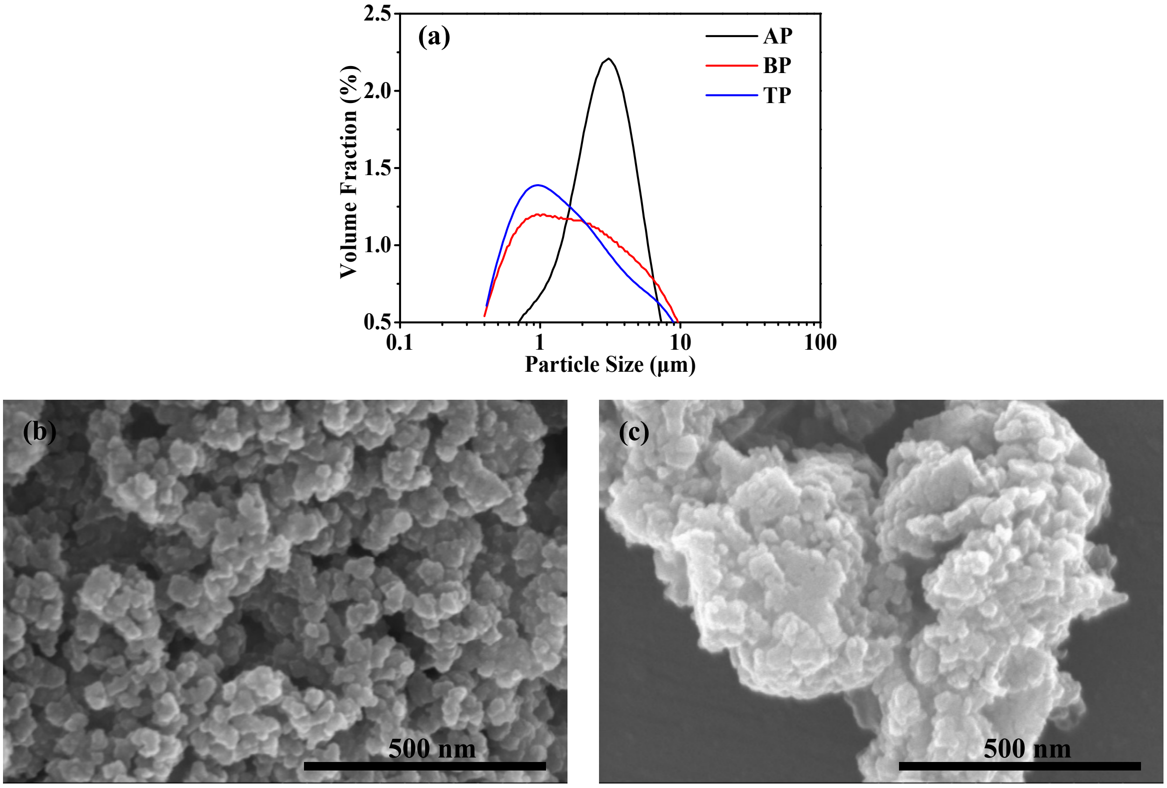

Figure S6. **(a)** DLS of AP, BP and TP; **(b)** SEM of BP; **(c)** SEM of TP .

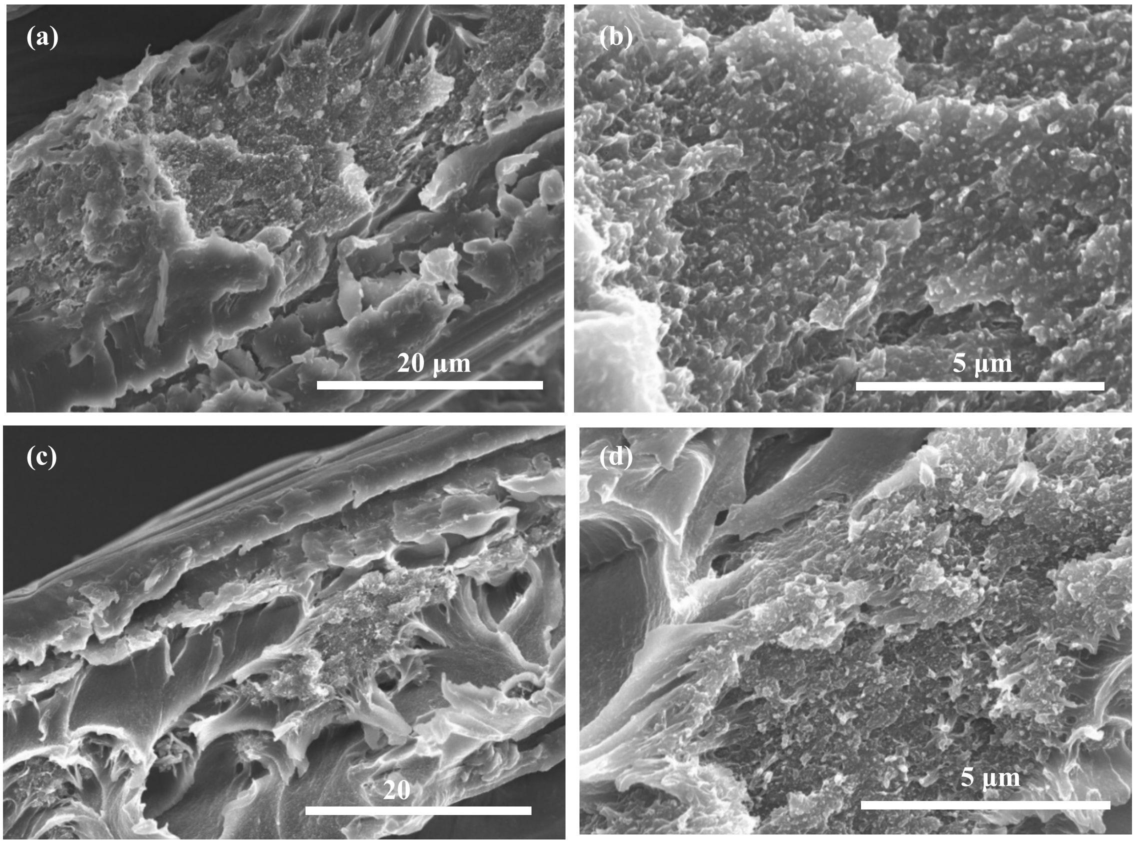

Figure S7. Cross-section SEM images of **(a, b)** 5BP/6FDA-Durene and amplification; **(c, d)** 5TP/6FDA-Durene and amplification

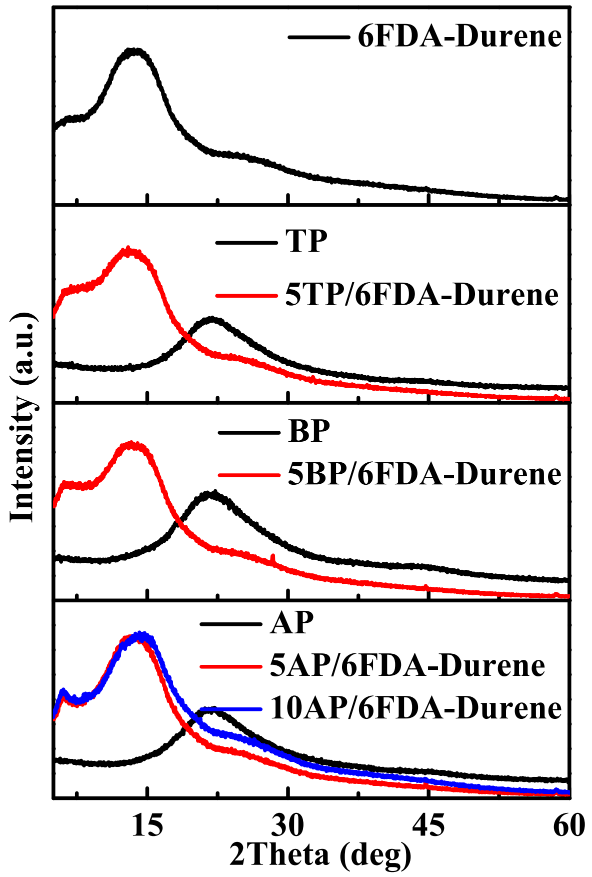

Figure S8. XRD patterns of microporous PIs, 6FDA-Durene and "all polyimide" MMMs.

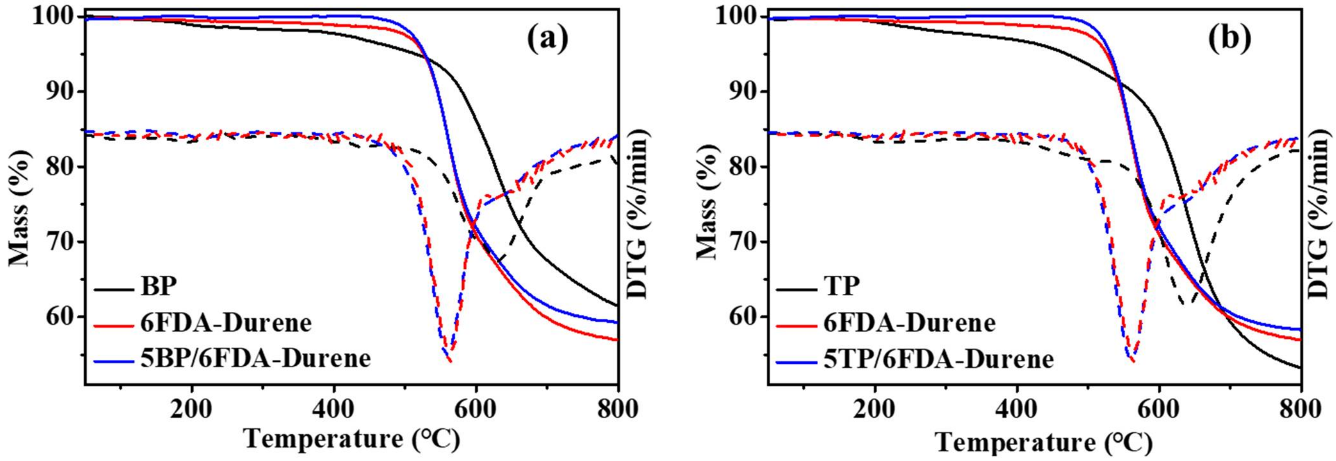

Figure S9. TGA (solid line) and DTG (dotted line) of **(a)** BP and the MMMs with BP loading; **(b)** TP and the MMMs with TP loading

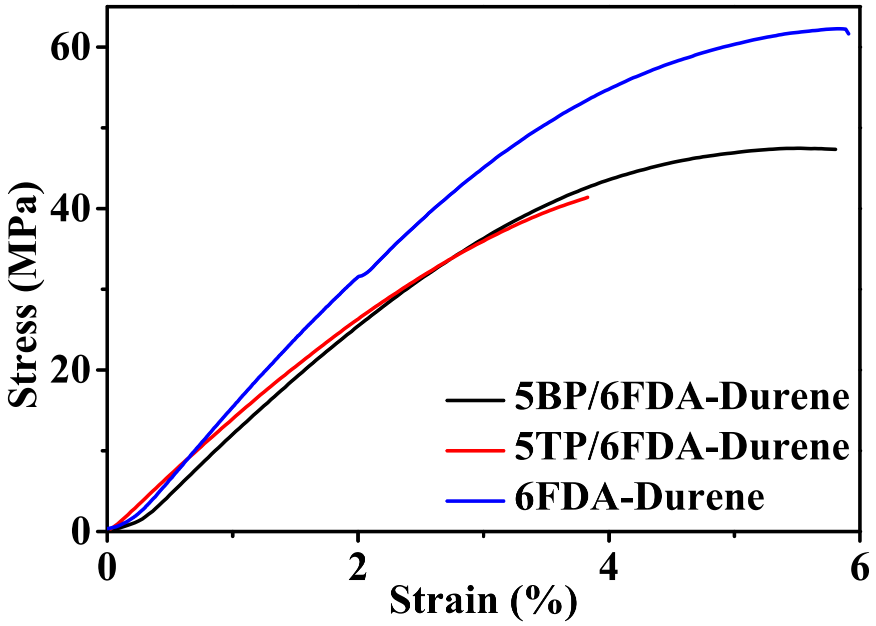

Figure S10. stress-strain curves of 5BP/6FDA-Durene, 5TP/6FDA-Durene and 6FDA-Durene

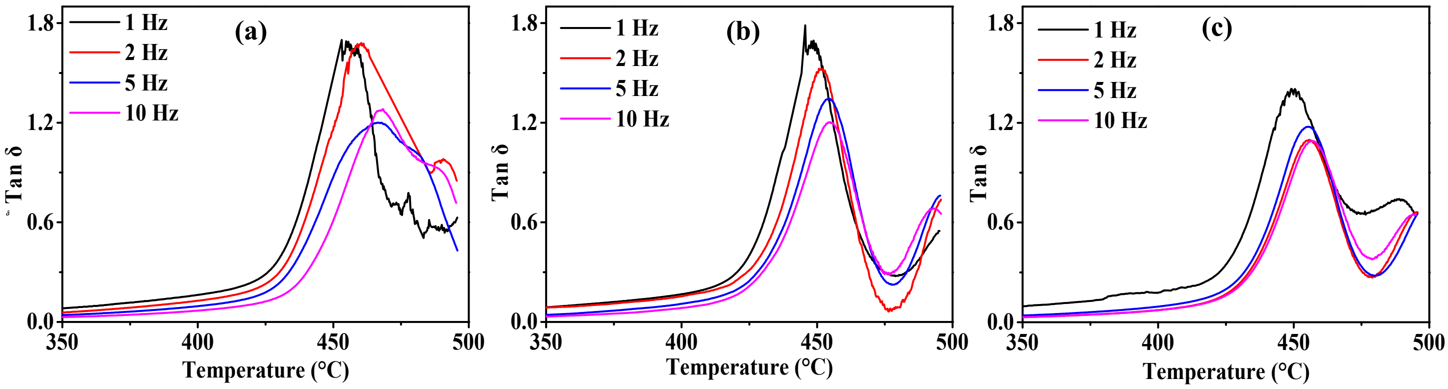

Figure S11. DMA curves of **(a)** 6FDA-Durene; **(b)** 5AP/6FDA-Durene; **(c)** 10AP/6FDA-Durene at 1 Hz, 2 Hz, 5 Hz, 10 Hz respectively

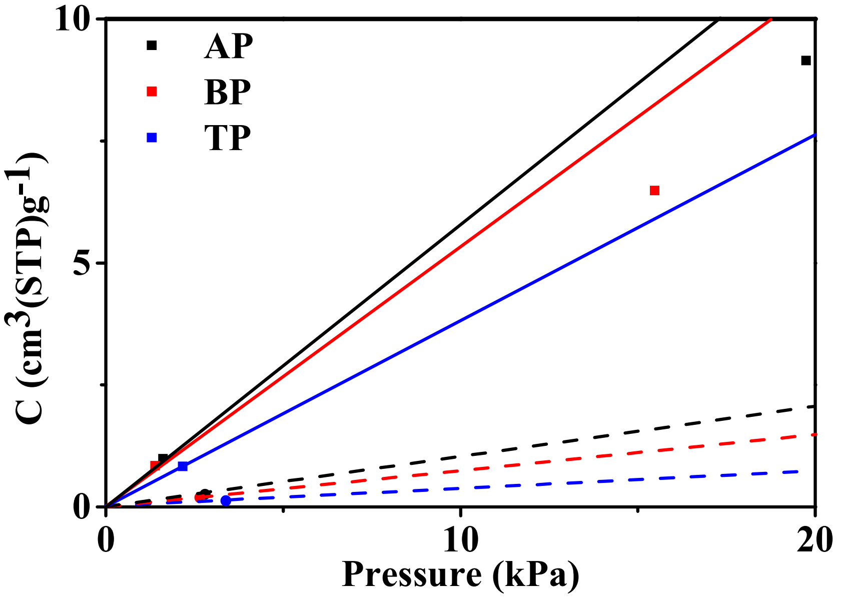

Figure S12. Henry fit for idea adsorption solution theory (IAST) of AP, BP and TP.

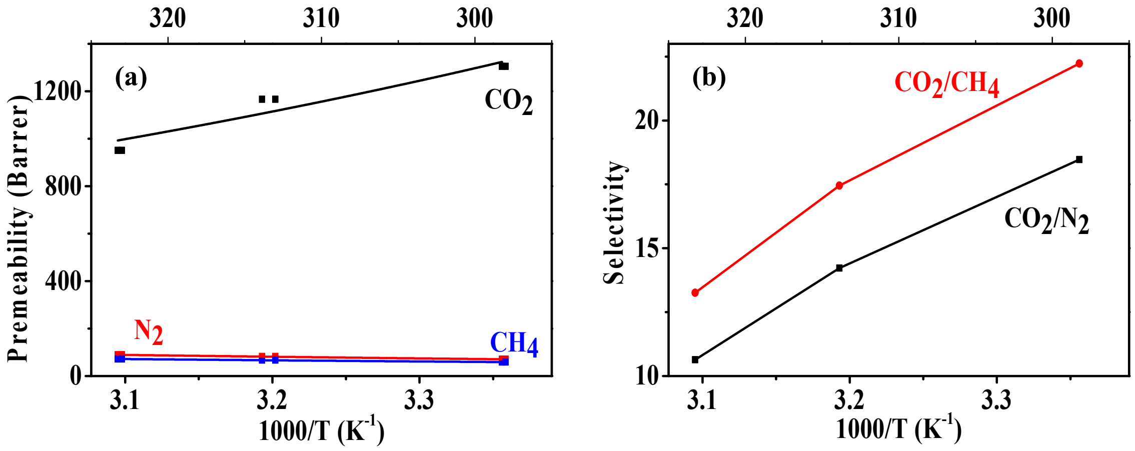

Figure S13. (a) Gas permeability and (b) ideal selectivity of 5AP/6FDA-Durene at 100 psi in different temperature.

Table S1. Assignments for **Figure. S1-S3**

	a	b	c	d	e	f	g	h	i
AP	125.2	137.1	165.8	127.2	122.5	134.2	146.9		
BP	125.2	135.5	158.4	130.5	130.5	128.1	136.4	137.5	120.3
TP	125.2	134.5	164.4	132.7	128.7	127.7	130.3	169.2	

Table S2. The ratio of P_{CO_2} of PI base MMMs to pristine PI membranes

Matrix	Filler	Filler content (wt%)	P_{CO_2} (Barrer)	P_{CO_2} increment Ratio	Ref
6FDA-	/		500.17	1	This work

Durene	AP	5	1291.13	2.58	
	AP	10	959.16	1.92	
	BP	5	1047.43	2.09	
	TP	5	753.07	1.51	
	/	0	805	1	
6FDA-Durene	ZIF-71 (30 nm)	21.2	2560	3.18	[1]
	ZIF-71 (200 nm)	21.1	2744	3.41	
	ZIF-71 (600 nm)	19.3	1656	2.06	
	/	0	625	1	
	NH2-MIL-101	10	1365	2.18	
	CNT	5	730	1.17	
6FDA-Durene	CNT	10	1100	1.76	[2]
	CNT	15	1700	2.72	
	CNT-MIL	5	800	1.28	
	CNT-MIL	10	1100	1.76	
	CNT-MIL	15	1625	2.60	
	/	0	800	1	
6FDA-Durene	HKUST-1	10	1300	1.63	[3]
	un-HKUST-IL	10	400	0.5	
	HKUST-IL	10	1100	1.38	
	/	0	1280	1	
	PSM-MOF	2.5	1310	1.02	
	PSM-MOF	5	1340	1.05	
	PSM-MOF	7.5	1370	1.07	
	PSM-MOF	10	1400	1.09	
	PSM-MOF	15	1470	1.15	
6FDA-Durene	PSM-MOF	20	1540	1.20	[4]
	PSM-MOF	25	1640	1.28	
	PSM-MOF	30	1710	1.34	
	PSM-MOF	35	1810	1.41	
	PSM-MOF	40	1890	1.48	
	PSM-MOF	100	3515	2.75	
	UiO-66-NH2	15	1990	1.55	
	UiO-66-NH2	30	3260	2.55	
6FDA-Durene	/	0	1143	1	[5]
	ZIF-71	10	2049	1.79	
	/	0	300	1	
6FDA-Durene	PgC5Cu	1	310	1.03	[6]
	PgC5Cu	2	360	1.2	
	PgC5Cu	3	410	1.37	
	/	0	455.2	1	
6FDA-Durene	NaX	5	620	1.36	[7]
	NaX	10	780	1.71	
	NaX	15	891.3	1.96	

	NaX	20	1000	2.20	
	[Cu(6L)]2+@13X	5	891.3	1.96	
	[Cu(6L)]2+@13X	10	960	2.11	
	[Cu(6L)]2+@13X	15	1034.1	2.27	
	[Cu(6L)]2+@13X	20	1060	2.33	
6FDA-Durene	/	0	626	1	[8]
	Ni(dobdc)	21	1035	1.65	
6FDA-Durene	/	0	959	1	[9]
	ZIF-71	10	1539	1.60	
6FDA-Durene	/	0	669.12	1	[10]
	ZIF-67	10	779.51	1.16	
	ZIF-67	20	1529.86	2.29	
	ZIF-67-IL1	10	779.01	1.16	
	ZIF-67-IL1	20	1254.64	1.88	
	ZIF-67-IL2	10	1030.96	1.54	
	ZIF-67-IL2	20	1426.12	2.13	
	ZIF-67-IL3	10	672.79	1.01	
	ZIF-67-IL3	20	889.87	1.33	
6FDA-Durene	/	0	460	1	[11]
	Zeolite T	0.5	700	1.52	
	Zeolite T	1	843.6	1.83	
	Zeolite T	3	700	1.52	
	Zeolite T	5	620	1.35	
	Zeolite T	7	390	0.85	
	Zeolite T	10	380	0.83	
	Zeolite T	15	370	0.80	
6FDA-DAM	/	0	546	1	[12]
	nano Mg2(dobpdc)	23	782	1.43	
	nano mmen-Mg2(dobpdc)	23	1056	1.93	
6FDA-DAM	/	0	444	1	[13]
	PPN	15	595	1.34	
	PPN	30	710	1.60	
6FDA-DAM	/	0	970	1	[14]
	HKUST-1	10	1450	1.49	
	HKUST-1	20	1690	1.74	
	HKUST-1	30	2360	2.43	
	branch HKUST-1	10	1600	1.65	
	branch HKUST-1	20	1920	1.98	
	branch HKUST-1	30	2480	2.56	
6FDA-DAM	/	0	420	1	[6]
	PgC5Cu	1	400	0.95	
	PgC5Cu	2	580	1.38	

6FDA-DAM	PgC5Cu	3	480	1.14	[15]
	/	0	590	1	
	ns-CuBDC	2	570	0.97	
	ns-CuBDC	4	430	0.73	
6FDA-DAM	/	0	518	1	[8]
	Ni(dobdc)	23	715	1.38	
6FDA-DAM	/	0	570	1	[16]
	G1NP	25	1050	1.84	
	G2NP	25	1920	3.37	
6FDA-DAM	/	0	1000	1	[17]
	ZMOF	20	1600	1.6	
6FDA-DAM	/	0	540	1	[18]
	3D-COF	10	850	1.57	
	3D-COF	15	1280	2.37	
	2D-COF	10	640	1.19	
6FDA-DAM	/	0	380	1	[19]
	NH2-MIL-53(Al) (NP)	5	440	1.16	
	NH2-MIL-53(Al) (NP)	10	480	1.26	
	NH2-MIL-53(Al) (NP)	15	600	1.58	
	NH2-MIL-53(Al) (NP)	20	700	1.84	
Matrimid	/	0	8.2	1	
	NH2-MIL-53(Al) (NP)	8	9.5	1.16	
	NH2-MIL-53(Al) (NP)	16	7.8	0.95	
	NH2-MIL-53(Al) (NR)	8	8	0.98	
	NH2-MIL-53(Al) (NP)	16	7.8	0.95	
	NH2-MIL-53(Al) (MN)	8	8	0.98	
	NH2-MIL-53(Al) (MN)	16	6.5	0.79	
Matrimid	/		6.82	1	[20]
	MMIF	10	8.1	1.19	
	MMIF	20	8.65	1.27	
matrimid	/	0	8.6	1	[13]
	PPN	15	20	2.33	
	PPN	30	61	7.09	
matrimid	/	0	9.55	1	[8]

	Ni(dobdc)	23	9.31	0.97	
matrimid	/	0	8	1	[21]
	UiO-66-NH2	12	18	2.25	
	UiO-66-NH2	23	24	3	
	UiO-66-NH2	40	27.5	3.44	
	UiO-66-NH2-Ipa	12	20	2.5	
	UiO-66-NH2-Ipa	23	29	3.63	
	UiO-66-NH2-Ipa	40	32	4	
	UiO-66-NH2-Ic	23	22.5	2.81	
	UiO-66-NH2-Isa	23	20	2.5	
Matrimid	/	0	10	1	[22]
	CMS	17	10.3	1.03	
	CMS	19	10.6	1.06	
	CMS	33	11.5	1.15	
	CMS	36	12.6	1.26	
matrimid	/	0	7.6	1	[23]
	PPI-1	20	10.3	1.36	
	PPI-2	10	11.3	1.49	
	PPI-2	20	16.8	2.21	
	PPI-3	20	20.9	2.75	
matrimid	/	0	5.78	1	[24]
	b-CuBDC	7.9	5.21	0.90	
	ns-CuBDC	7.9	4.09	0.70	
	ns-CuBDC	8.3	5.03	0.87	
	ns-CuBDC	1.7	5.38	0.93	
	ns-Cu(2,6-NDC)	7.6	6.28	1.09	
6FDA-ODA	/	0	20	1	[25]
	Cd-6F	10	38	1.9	
	Cd-6F	10	30	1.5	
	Cd-6F	10	41	2.05	
6FDA-6FpDA	/	0	71	1	[13]
	PPN	15	131	1.85	
	PPN	30	190	2.68	
TB-polyimide	/	0	285	1	[26]
	ZIF-8	7	560	1.96	
	ZIF-8	20	896	3.14	
	ZIF-8	30	1437	5.04	
	PDZIF-8	7	380	1.33	
	PDZIF-8	20	702	2.46	
	PDZIF-8	30	1056	3.71	
6FDA-DAT	/	0	55.8	1	[8]
	Ni(dobdc)	15	63.9	1.15	
6FBI	/	0	9	1	[27]
	ZIF-8	10	11.8	1.31	

	ZIF-8	20	20.3	2.26	
	ZIF-8	25	25.4	2.82	
	ZIF-8	30	50.9	5.65	
	/	0	1.45	1	
Ulten	CMS	16	2.51	1.73	[22]
	CMS	20	2.9	2	
	CMS	35	4.48	3.09	
	/	0	224	1	
6FDA-IMM	PPI-1	20	202	0.90	[23]
	PPI-2	20	270	1.21	
	PPI-3	20	289	1.29	

Table S3. Henry's law region fit for IAST of microporous PIs.

	Initial slope (CO_2)	Initial slope (CH_4)	selectivity
AP	0.335	0.083	4.02
BP	0.309	0.068	4.53
TP	0.196	0.036	5.43

Table S4. 5AP/6FDA-Durene activation energy (E_P) of permeation for CO_2, N_2 and CH_4

	E_P (kJ·mol^{-1})		
	CO_2	N_2	CH_4
5AP	-9.2	7.5	6.4

REFERENCES

1. Japip, S.; Xiao, Y.; Chung, T.-S. Particle-Size Effects on Gas Transport Properties of 6FDA-Durene/ZIF-71 Mixed Matrix Membranes. *Ind. Eng. Chem. Res.* **2016**, *55*, 9507-9517.
2. Lin, R.; Ge, L.; Liu, S.; Rudolph, V.; Zhu, Z. Mixed-Matrix Membranes with Metal–Organic Framework-Decorated CNT Fillers for Efficient CO_2 Separation. *ACS Appl. Mater. Interfaces* **2015**, *7*, 14750-14757.
3. Lin, R.; Ge, L.; Diao, H.; Rudolph, V.; Zhu, Z. Ionic Liquids as the MOFs/Polymer Interfacial Binder for Efficient Membrane Separation. *ACS Appl. Mater. Interfaces* **2016**, *8*, 32041-32049.
4. Qian, Q.; Wu, A.X.; Chi, W.S.; Asinger, P.A.; Lin, S.; Hypsher, A.; Smith, Z.P. Mixed-Matrix Membranes Formed from Imide-Functionalized UiO-66-NH_2 for Improved Interfacial Compatibility. *ACS Appl. Mater. Interfaces* **2019**, *11*, 31257-31269.
5. Japip, S.; Liao, K.S.; Chung, T.S. Molecularly Tuned Free Volume of Vapor Cross-Linked 6FDA-Durene/ZIF-71 MMMs for H_2/CO_2 Separation at 150 degrees C. *Adv. Mater.* **2017**, *29*.
6. Wang, H.; Zhang, K.; Ho Li, J.P.; Huang, J.; Yuan, B.; Zhang, C.; Yu, Y.; Yang, Y.; Lee, Y.; Li, T. Engineering Plasticization Resistant Gas Separation Membranes Using Metal–Organic Nanocapsules. *Chem. Sci.* **2020**, *11*, 4687-4694.
7. Mashhadikhan, S.; Moghadassi, A.; Ebadi Amooghin, A.; Sanaeepur, H. Interlocking a Synthesized Polymer and Bifunctional Filler Containing the Same Polymer's Monomer for Conformable Hybrid Membrane Systems. *J. Mater. Chem. A* **2020**, *8*, 3942-3955.
8. Bachman, J.E.; Long, J.R. Plasticization-Resistant Ni_2(dobdc)/Polyimide Composite Membranes for

the Removal of CO_2 from Natural Gas. *Energ. Environ. Sci.* **2016**, *9*, 2031-2036.
9. Japip, S.; Liao, K.-S.; Xiao, Y.; Chung, T.-S. Enhancement of Molecular-Sieving Properties by Constructing Surface Nano-Metric Layer via Vapor Cross-Linking. *J. Membrane Sci.* **2016**, *497*, 248-258.
10. Vu, M.-T.; Lin, R.; Diao, H.; Zhu, Z.; Bhatia, S.K.; Smart, S. Effect of Ionic Liquids (ILs) on MOFs/Polymer Interfacial Enhancement in Mixed Matrix Membranes. *J. Membrane Sci.* **2019**, *587*.
11. Jusoh, N.; Yeong, Y.F.; Lau, K.K.; M. Shariff, A. Enhanced Gas Separation Performance Using Mixed Matrix Membranes Containing Zeolite T and 6FDA-Durene Polyimide. *J. Membrane Sci.* **2017**, *525*, 175-186.
12. Maserati, L.; Meckler, S.M.; Bachman, J.E.; Long, J.R.; Helms, B.A. Diamine-Appended Mg_2(dobpdc) Nanorods as Phase-Change Fillers in Mixed-Matrix Membranes for Efficient CO_2/N_2 Separations. *Nano Lett.* **2017**, *17*, 6828-6832.
13. Aguilar-Lugo, C.; Suárez-García, F.; Hernández, A.; Miguel, J.A.; Lozano, Á.E.; de la Campa, J.G.; Álvarez, C. New Materials for Gas Separation Applications: Mixed Matrix Membranes Made from Linear Polyimides and Porous Polymer Networks Having Lactam Groups. *Ind. Eng. Chem. Res.* **2019**, *58*, 9585-9595.
14. Chi, W.S.; Sundell, B.J.; Zhang, K.; Harrigan, D.J.; Hayden, S.C.; Smith, Z.P. Mixed-Matrix Membranes Formed from Multi-Dimensional Metal-Organic Frameworks for Enhanced Gas Transport and Plasticization Resistance. *ChemSusChem* **2019**, *12*, 2355-2360.
15. Yang, Y.; Goh, K.; Wang, R.; Bae, T.H. High-Performance Nanocomposite Membranes Realized by Efficient Molecular Sieving with CuBDC Nanosheets. *Chem. Commun.* **2017**, *53*, 4254-4257.
16. Hasebe, S.; Aoyama, S.; Tanaka, M.; Kawakami, H. CO_2 Separation of Polymer Membranes Containing Silica Nanoparticles with Gas Permeable Nano-Space. *J. Membrane Sci.* **2017**, *536*, 148-155.
17. Liu, G.; Labreche, Y.; Chernikova, V.; Shekhah, O.; Zhang, C.; Belmabkhout, Y.; Eddaoudi, M.; Koros, W.J. Zeolite-like MOF Nanocrystals Incorporated 6FDA-Polyimide Mixed-Matrix Membranes for CO_2/CH_4 Separation. *J. Membrane Sci.* **2018**, *565*, 186-193.
18. Yang, Y.; Goh, K.; Weerachanchai, P.; Bae, T.-H. 3D Covalent Organic Framework for Morphologically Induced High-Performance Membranes with Strong Resistance Toward Physical Aging. *J. Membrane Sci.* **2019**, *574*, 235-242.
19. Sabetghadam, A.; Seoane, B.; Keskin, D.; Duim, N.; Rodenas, T.; Shahid, S.; Sorribas, S.; Le Guillouzer, C.; Clet, G.; Tellez, C., et al. Metal Organic Framework Crystals in Mixed-Matrix Membranes: Impact of the Filler Morphology on the Gas Separation Performance. *Adv. Funct. Mater.* **2016**, *26*, 3154-3163.
20. Ahmadi, M.; Tas, E.; Kilic, A.; Kumbaraci, V.; Talinli, N.; Ahunbay, M.G.; Tantekin-Ersolmaz, S.B. Highly CO_2 Selective Microporous Metal-Imidazolate Framework-Based Mixed Matrix Membranes. *ACS Appl. Mater. Interfaces* **2017**, *9*, 35936-35946.
21. Venna, S.R.; Lartey, M.; Li, T.; Spore, A.; Kumar, S.; Nulwala, H.B.; Luebke, D.R.; Rosi, N.L.; Albenze, E. Fabrication of MMMs with Improved Gas Separation Properties Using Externally-Functionalized MOF Particles. *J. Mater. Chem. A* **2015**, *3*, 5014-5022.
22. Vu, D.Q.; Koros, W.J.; Miller, S.J. Mixed Matrix Membranes Using Carbon Molecular Sieves - I. Preparation and Experimental Results. *J. Membrane Sci.* **2003**, *211*, 311-334.
23. Rangel Rangel, E.; Maya, E.M.; Sánchez, F.; de Abajo, J.; de la Campa, J.G. Gas Separation Properties of Mixed-Matrix Membranes Containing Porous Polyimides Fillers. *J. Membrane Sci.* **2013**, *447*, 403-412.
24. Rodenas, T.; Luz, I.; Prieto, G.; Seoane, B.; Miro, H.; Corma, A.; Kapteijn, F.; Llabres, I.X.F.X.; Gascon, J. Metal-Organic Framework Nanosheets in Polymer Composite Materials for Gas Separation. *Nat. Mater.* **2015**, *14*, 48-55.
25. Lin, R.; Ge, L.; Hou, L.; Strounina, E.; Rudolph, V.; Zhu, Z. Mixed Matrix Membranes with Strengthened MOFs/Polymer Interfacial Interaction and Improved Membrane Performance. *ACS Appl. Mater. Interfaces* **2014**, *6*, 5609-5618.
26. Wang, Z.; Wang, D.; Zhang, S.; Hu, L.; Jin, J. Interfacial Design of Mixed Matrix Membranes for Improved Gas Separation Performance. *Adv. Mater.* **2016**, *28*, 3399-3405.
27. Fan, Y.; Yu, H.; Xu, S.; Shen, Q.; Ye, H.; Li, N. Zn(II)-Modified Imidazole Containing Polyimide/ZIF-8 Mixed Matrix membranes for Gas Separations. *J. Membrane Sci.* **2020**, *597*.
